# Introducing PIGMO, a novel PIGmented MOuse model of Parkinson’s disease

**DOI:** 10.1038/s41531-026-01289-9

**Published:** 2026-02-11

**Authors:** Julia Chocarro, Sergio Marana, Maria Espelosin, Alberto J. Rico, Goiaz Ariznabarreta, Elena Lorenzo-Ramos, Mario M. Ilarduya, Ruben Hernandez-Alcoceba, Miquel Chillón, Miquel Vila, Jeffrey H. Kordower, Anthony H. V. Schapira, Ana Garcia-Osta, Maria del Mar Cuadrado-Tejedor, Jose L. Lanciego

**Affiliations:** 1 CNS Gene Therapy Program, Center for Applied Medical Research (Cima), University of Navarra, Pamplona, Spain; 2 Centro de Investigación Biomédica en Red de Enfermedades Neurodegenerativas (Ciberned-ISCIII), Madrid, Spain; 3 Aligning Science Across Parkinson’s (ASAP) Collaborative Research Network, Chevy Chase, MD USA; 4 Institut des Neurociences, Department of Biochemistry and Molecular Biology, University Autónoma de Barcelona, Barcelona, Spain; 5 Val d’Hebron Research Institute, Barcelona, Spain; 6 Institució Catalana de Recerca i Estudis Avançats (ICREA), Barcelona, Spain; 7 ASU-Banner Neurodegenerative Disease Research Center, Arizona State University, Tempe, AZ USA; 8 Department of Clinical Neurosciences, University College London (UCL) Institute of Neurology, Royal Free Campus, London, UK

**Keywords:** Biological techniques, Diseases, Neurology, Neuroscience

## Abstract

There is a pressing need for the development, characterization, and standardization of animal models of Parkinson’s disease (PD) that properly mimic the cardinal features of this disorder, comprising both the motor phenotype and neuropathological signatures. In the past few years, animal modeling has moved from neurotoxin-based approaches toward viral vectors carrying a given genetic payload of interest. Here, to induce pigmentation of the mouse brain upon systemic delivery, we took advantage of a modified adeno-associated viral vector capsid engineered to bypass the blood-brain barrier and coding for the human tyrosinase gene (AAV9-P31-*hTyr*). Obtained results revealed an ongoing pigmentation of catecholaminergic centers related to the pathophysiology of PD, such as the substantia nigra pars compacta, ventral tegmental area, and locus coeruleus. Moreover, pigmented dopaminergic neurons exhibited Lewy body-like intracytoplasmic inclusions, a progressive nigrostriatal degeneration, and a time-dependent PD motor phenotype. The bilateral pigmented model of PD generated in this way does not require stereotactic surgery for viral vector delivery, opening up unprecedented possibilities for preclinical testing of therapeutic candidates designed to reduce disease progression rates.

## Introduction

In the past few decades, the field of Parkinson’s disease (PD) has witnessed the introduction of novel generations of animal models that played an instrumental role in advancing the current understanding of the underlying physiopathological mechanisms and the development of therapeutic candidates. However, important limitations remain to be addressed, since by definition, any given model only replicates a small fraction of the inherent complexity of the clinicopathological spectrum of PD, and therefore, in most cases, models themselves do not accurately represent the real disease^1–6^. Nevertheless, it is undeniable that available animal models of PD have paved the way for the implementation of pharmacological and neurosurgical therapies, providing patients with a clear benefit, both in terms of symptomatic alleviation and improvement of quality of life. Despite all the successes achieved with symptomatic treatments, disease-modifying therapeutics tested in existing models repeatedly failed when translated into clinical trials, and thus, rather than the putative candidate, it is very likely that the main limitations lie within the animal models themselves^2,7^.

Although rodent models based on viral vector-mediated expression of different alpha-synuclein species (wild type or mutated) have provided promising results by overcoming existing barriers in earlier generations of neurotoxin-based models and transgenic mice, these models also have their inherent limitations^3,8^. To mention a few, alpha-synuclein expression obtained this way is far beyond physiological levels and often results in a variable degree of cell loss and nigral pathology. Choices made regarding the viral serotype, promoter, and delivery route directly influence the obtained phenotype; therefore, a better standardization is still needed to ensure appropriate outcomes and reproducibility^7^.

The introduction of adeno-associated viral vectors (AAVs) coding for the human tyrosinase gene (*hTyr*) as tools for modeling PD in rats and non-human primates^9,10^ has opened a new avenue in the field of animal modeling. The AAV-mediated enhanced expression of tyrosinase leads to a time-dependent accumulation of neuromelanin (NMel), a progressive death of pigmented dopaminergic neurons, the presence of Lewy body-like intracytoplasmic inclusions (LBL) made of endogenous alpha-synuclein, and a pro-inflammatory environment, together with a motor phenotype (the latter being observed only in rats and not in macaques). Moreover, a transgenic mouse based on the constitutive expression of tyrosinase has been made available recently^11^. Animal models of PD obtained in these ways are based on earlier postulates linking NMel pigmentation and dopaminergic cell vulnerability^12–14^ as well as on accumulated clinical evidence showing a bi-directional relationship between the incidence of PD and melanoma, a cancer of skin melanocytes^15^. Indeed, alpha-synuclein has been detected in cultured melanoma cells and tissues derived from patients with melanoma^16^. Clinical associations between Gaucher disease and melanoma have also been reported^17,18^. In this regard, mutations in the GBA1 gene (encoding the lysosomal enzyme glucocerebrosidase) currently represent the main genetic risk factor for PD^19^.

Since mice are the most commonly used experimental laboratory animals, we took advantage of an AAV serotype 9 capsid engineered to bypass the blood-brain barrier (BBB) upon intravenous administration^20,21^. BBB transcytosis of AAV9-P31 capsid variant is mediated by the carbonic anhydrase IV receptor, a protein expressed on the surface of endothelial cells^22^. The intravenous administration of AAV9-P31 coding for the *hTyr* gene resulted in a novel rodent model (termed PIGMO model) mimicking the known motor and neuropathological phenotypes of human PD with unprecedented accuracy.

## Results

### Time-dependent accumulation of neuromelanin

The systemic delivery of AAV9-P31-*hTyr* resulted in a time-dependent bilateral pigmentation of brain catecholaminergic centers of the mouse brain mimicking pigmentation patterns observed in NMel transgenic mice^11^. The conducted study was focused on the substantia nigra pars compacta (SNpc), the ventral tegmental area (VTA), and the locus coeruleus (LC), all these nuclei related to the pathophysiology of PD, according to the Braak hypothesis that postulates a clinico-pathological correlate of disease progression^23,24^.

**Fig. 1:**
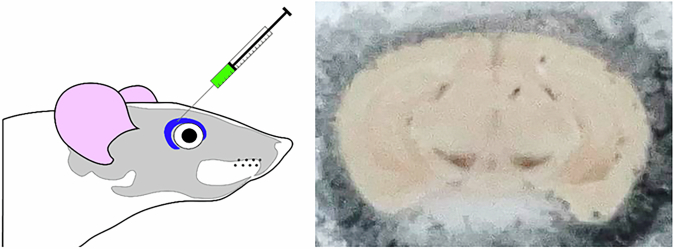
Systemic delivery of viral vectors. Schematic representation of the procedure for viral vector injections into the retro-ocular venous sinus of mice, together with a macroscopic visualization of the obtained neuromelanin pigmentation in the substantia nigra pars compacta (SNpc) and ventral tegmental area (VTA) as observed in a mouse injected with AAV9-P31-*hTyr* 4 months post-viral delivery.

**Fig. 2:**
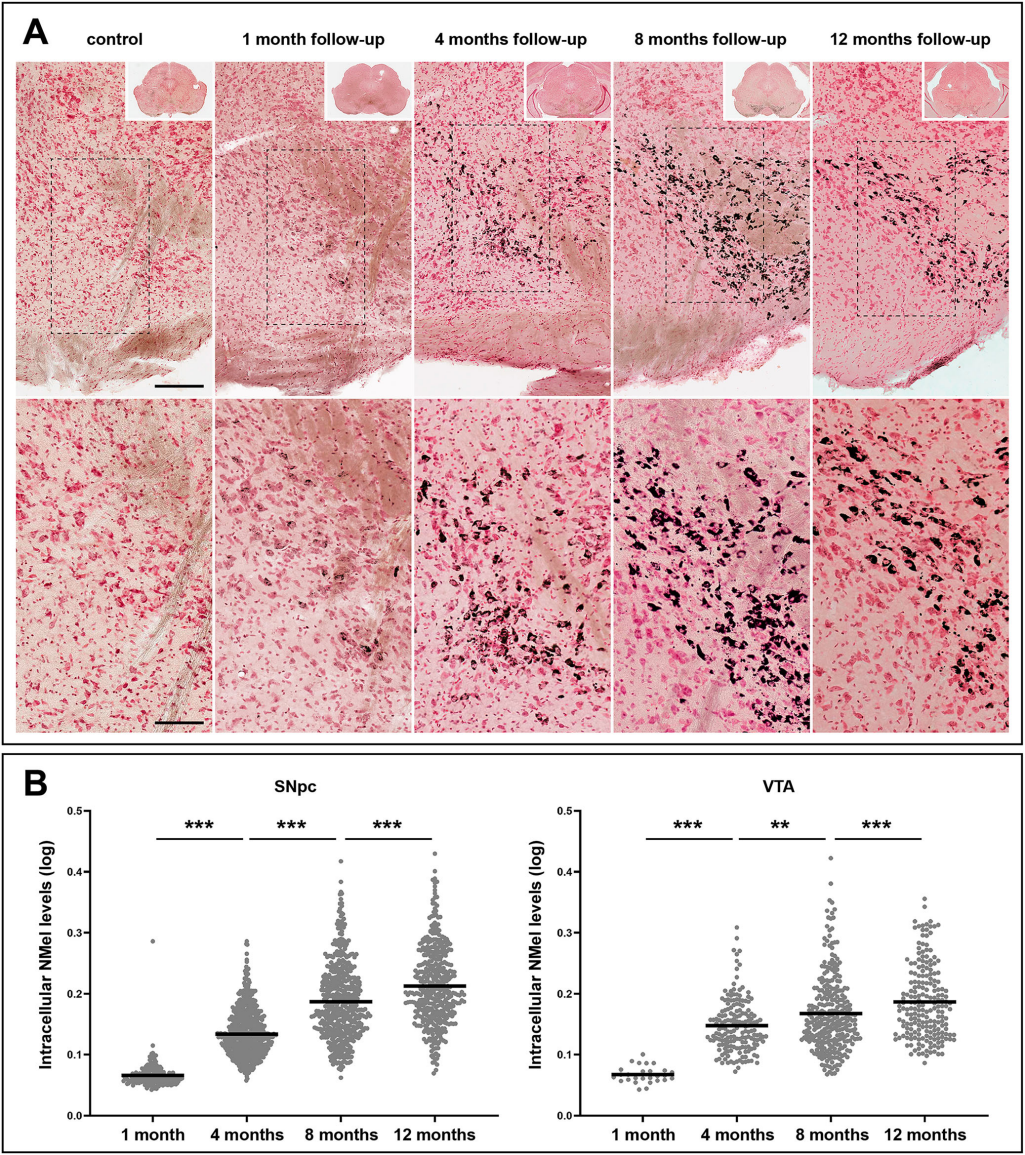
Time-dependent pigmentation of SNpc and VTA neurons. **A** Progressive accumulation of neuromelanin (NMel). A weak pigmentation was first observed one month post-injection of AAV9-P31-*hTyr*. Accumulation of NMel increased over time, as shown in representative photomicrographs taken 4, 8, and 12 months of follow-up. Scale bars, 100 μm for low-magnification images and 200 μm in insets. **B** Boxplots showing intracellular neuromelanin levels at the single-cell level for all experimental groups in the SNpc and VTA. In both structures, a significant time-dependent increase in intracellular pigmentation was observed. Within each experimental group (made of three biological replicates each), pigmentation values are pooled together to obtain a mean value (Table 2 shows the number of neurons used for quantifying intracellular neuromelanin levels in each experimental subject). Values are mean ± SEM, nested ANOVA test with time as a fixed factor, and mice nested with a fixed factor. At the SNpc level, *p* < 0.001 (4 vs. 1 month, 8 vs. 4 months, and 12 vs. 8 months). *p* < 0.01 when comparing different 4 vs. 8 months at the level of the VTA.

Initial traces of intracellular NMel accumulation in SNpc neurons were observed as early as 1 month post-delivery of AAV9-P31-*hTyr*, with a moderate number of weakly pigmented neurons. By contrast, the same follow-up period resulted in a small number of pigmented neurons in the VTA, with minimal levels of intracellular NMel. Accumulation of NMel gradually increased over time, reaching levels high enough to enable a direct macroscopic visualization of the pigmented SNpc and VTA nuclei beyond 4 months post-viral vector deliveries (Fig. 1). In parallel to the increase in neuromelanized SNpc neurons, intracellular pigmentation levels also rose over time. In other words, the number and NMel levels of pigmented neurons followed a time-dependent pattern (Fig. 2). That said, although intracellular pigmentation levels within SNpc neurons still increased from 8 to 12 months post-viral delivery, the total number of neuromelanized neurons showed some decline, likely reflecting a more pronounced nigrostriatal degeneration at 12 months. Moreover, a tier-specific pigmentation of dopaminergic neurons was observed upon the systemic delivery of AAV9-P31-*hTyr* in mice. Neurons in the ventral tier of the SNpc that are positive for aldehyde dehydrogenase type 1a1 (Aldh1a1+) are those accumulating NMel, whereby calbindin-positive neurons (CB+) in the dorsal tier never became pigmented (Supplementary Fig. 2).

**Fig. 3:**
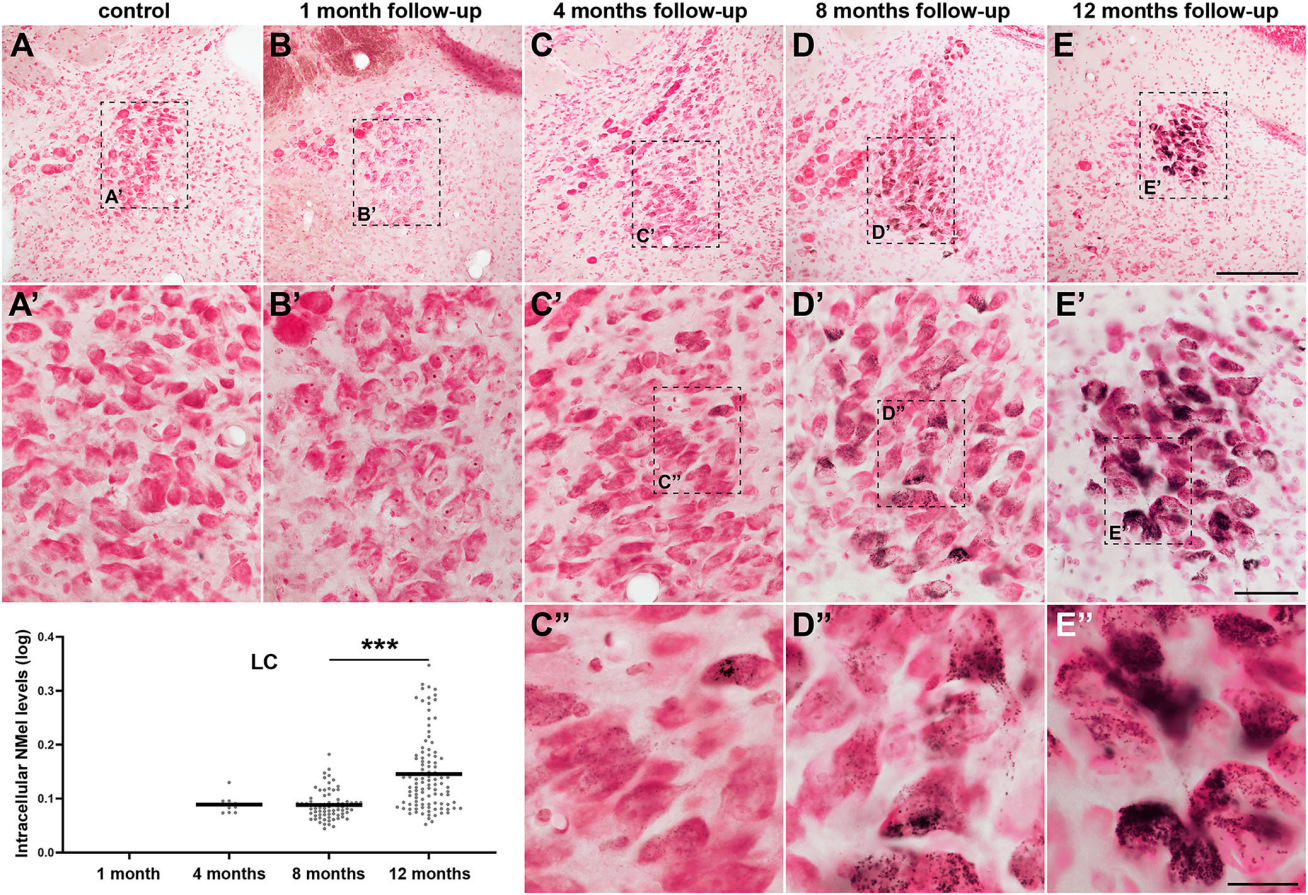
Time-dependent pigmentation of LC neurons. Noradrenergic neurons of the LC became progressively pigmented following the delivery of AAV9-P31-*hTyr* viral vector, although to a lower level than dopaminergic neurons in the SNpc and VTA. By 12 months of follow-up, accumulation of NMel is similar to SNpc and VTA neurons 4 months post-viral injection. Boxplots indicate values for intracellular NMel pigmentation. Within each experimental group (made of three biological replicates each), pigmentation values are pooled together to obtain a mean value (Table 2 shows the number of neurons used for quantifying intracellular neuromelanin levels in each experimental subject). Values are mean ± SEM, nested ANOVA test with time as a fixed factor, and mice nested with a fixed factor. *p* < 0.001 between 8 and 12 months. Scale bars, 200 μm in panels **A**–**E**; 50 μm in **A**’–**E**’, and 20 μm in **C**” and **D**”.

Compared to the SNpc, neurons in the VTA showed a similar temporal pattern of gradual pigmentation, but to a lower magnitude. The number of neuromelanized VTA neurons and their intracellular pigmentation levels are time-dependent; however, the slope of said increase is far less pronounced than in the SNpc (Fig. 2). Regarding the LC, noradrenergic neurons appeared to be more resistant to pigmentation. Neuromelanized neurons in the LC were first detected 4 months post-systemic delivery of AAV9-P31-*hTyr*, and exhibited minimal traces of intracellular NMel accumulation. Although more neurons in the LC became pigmented over time, no differences were observed when comparing intracellular pigmentation levels between 4 and 8 months post-delivery. The highest accumulation of NMel in the LC was found with a follow-up period of 12 months (Fig. 3), reaching pigmentation levels only comparable to those observed in the SNpc and VTA with a survival time of 4 months (Figs. 2 and 3).

### Lewy body-like inclusions in pigmented neurons

**Fig. 4:**
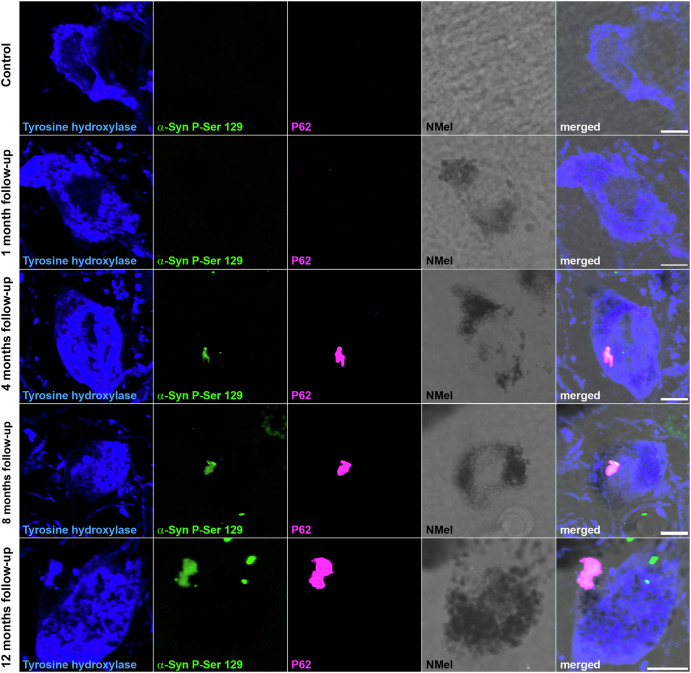
Intracytoplasmic aggregates in dopaminergic neurons. Intracellular inclusions within dopaminergic neurons of the SNpc (TH+; blue channel) followed a time-dependent pattern. Although a weak NMel accumulation is already observed one month post-delivery of AAV9-P31-*hTyr*, pigmentation levels are not high enough to induce Lewy body-like intracytoplasmic aggregates. Inclusions positive for phosphorylated alpha-synuclein (P-Ser 129; green channel) and P62 (purple channel) are observed in experimental groups with 4, 8, and 12 months of survival time, in parallel to the ongoing increase of pigmentation. Scale bars, 5 μm.

**Fig. 5:**
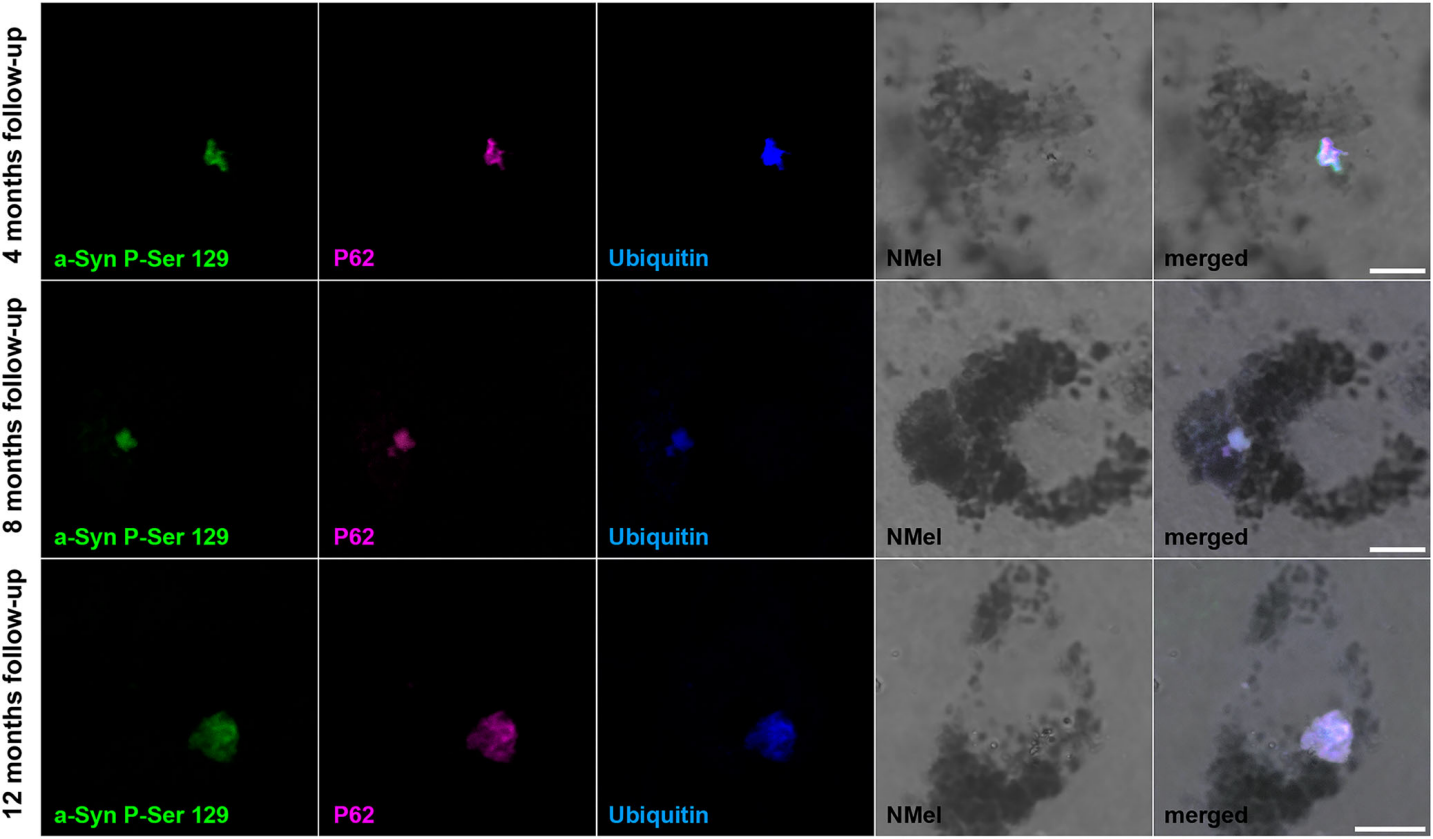
Markers of Lewy body-like inclusions in pigmented neurons of the SNpc. Triple immunofluorescent detection of Lewy body markers within pigmented neurons in the SNpc, comprising phosphorylated alpha-synuclein (P-Ser 129; green channel), P62 (purple), and ubiquitin (blue). As expected, intracytoplasmic inclusions were found in heavily neuromelanized neurons, bearing in mind that pigmentation beyond a given threshold is required to induce aggregation of endogenous alpha-synuclein in the form of Lewy bodies. Scale bars, 5 μm.

The presence of intracytoplasmic inclusions was analyzed at different time points post-systemic delivery of AAV9-P31-*hTyr*. In keeping with earlier data linking NMel levels beyond a given threshold as drivers inducing Lewy body-like pathology (LBL^9–11^), intracytoplasmic inclusions were observed in the SNpc of all animals with a follow-up period beyond 4 months (Fig. 4). Although pigmented neurons were first noticed one month post-injection of AAV9-P31-*hTyr*, NMel levels were not high enough to trigger the accumulation of endogenous alpha-synuclein in the form of LBL after such a short survival time. LBLs were first observed in dopaminergic neurons of the SNpc 4 months post-delivery of the BBB-penetrant viral vector encoding the *hTyr* gene. Although the total number of LBLs was not quantified, intracellular inclusions were more abundant in all animals sacrificed after 8 and 12 months post-viral deliveries. Observed inclusions were positive for traditional markers of LB pathology, such as PK-resistant phosphorylated alpha-synuclein (Ser129), P62, and ubiquitin (Fig. 5). Regarding the VTA, LBL pathology was most often observed after 8 and 12 months of follow-up. Finally, intracytoplasmic inclusions were only found in the LC 12 months post-viral delivery (Supplementary Fig. 3), likely reflecting lower pigmentation levels that typically characterize LC neurons, which are equivalent to the pigmentation levels found in the SNpc and VTA after a follow-up of 4 months (Figs. 2 and 3).

### Progressive nigrostriatal degeneration

In parallel to NMel accumulation and LBL burden, the observed dopaminergic cell loss in the SNpc and the corresponding decline of dopaminergic terminals in the striatum followed a time-dependent pattern.

**Fig. 6:**
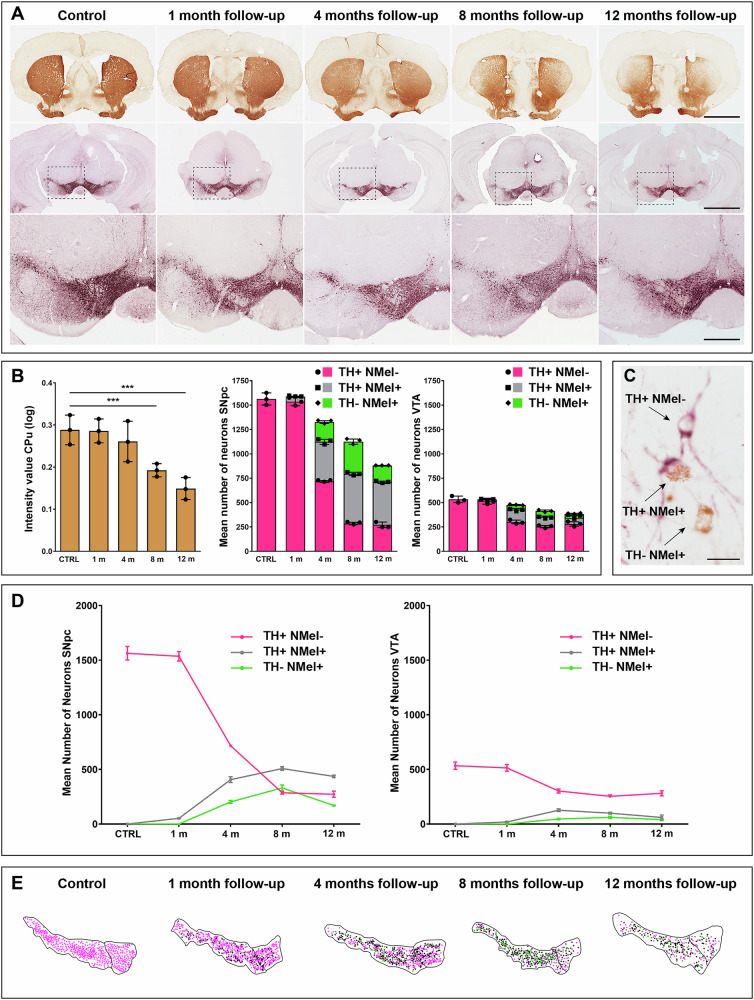
Progressive nigrostriatal lesion. The time-dependent NMel accumulation and the subsequent endogenous synucleinopathy triggered an ongoing lesion of the nigrostriatal pathway, both at origin (SNpc) and at destination (striatum). **A** Immunohistochemical detection of TH in the striatum (brown reaction product) and in the substantia nigra (purple peroxidase substrate) showing that nigrostriatal damage increased over time. A bilateral loss of TH+ terminals in dorsolateral striatal territories, reflecting progressive death of nigrostriatal-projecting neurons of the SNpc, was noticed, even at a low magnification. Scale bars, 2000 μm low-magnification images taken from the striatum and SNpc, and 500 μm in insets. **B** Histograms showing the progressive decline of TH+ terminals in the striatum and the corresponding phenotype-specific cell loss in the SNpc and VTA. The quantification of optical density for dopaminergic terminals in the striatum is represented as mean ± SEM, unpaired *t*-test. *p* < 0.001 between control animals and 8 months, and *p* < 0.0001 between controls and 12 months. Each experimental group comprises three biological specimens. **C** Photomicrograph taken from the SNpc in an experimental subject with 8 months of follow-up showing three distinctive neuronal phenotypes, namely non-pigmented TH+ neurons (TH+/ NMel−), neuromelanized TH+ neurons (TH+/ NMel+), and ghost cells (TH−/ NMel+). Scale bar, 20 μm. **D** Schematic representation of the dynamic changes observed over time across the three characteristic cellular phenotypes. In the SNpc, the main cell loss was observed between 1 and 4 months, and from 4 to 8 months. Beyond 8 months of survival time, the number of TH+/ NMel− reached a plateau, likely suggesting the presence of a small cellular reservoir made of non-pigmented neurons that are more resilient to degeneration. Moreover, pigmented dopaminergic neurons (TH+/ NMel+) and ghost cells (TH−/NMel+) followed a similar pattern, reaching a maximum peak at 8 months and with a moderate decline at 12 months. This decline is higher for ghost neurons, which are the most vulnerable ones. **E** Camera lucida drawings taken from a representative level of the SNpc and VTA across all experimental groups, showing the ongoing changes for all the different cellular phenotypes.

Optical densities for TH+ axon terminals in the striatum gradually declined over time (Fig. 6A and B). Compared to control animals, no differences were observed one month post-delivery of the viral vector. A minor decrease was first observed at 4 months (10.34% of reduction in optical density) without reaching statistical significance. In animals euthanized 8 months post-injection, a significant bilateral reduction in TH+ terminals was observed in dorsolateral territories of the striatum (34.48% of decline compared to control animals), further reaching a more severe reduction (50.12% on average) in animals with a follow-up of 12 months, where the loss of TH+ terminals in the dorsolateral striatum is far more pronounced.

The gradual damage of TH+ terminals in the striatum was in keeping with a similar pattern of progressive dopaminergic cell loss observed in the SNpc and, to a minor extent, in the VTA (Fig. 6). Compared to control specimens, the number of dopaminergic cells started to decline 4 months post-systemic deliveries of AAV9-P31-*hTyr* (16.67% of cell loss at 4 months), this pattern of cell loss increasing over time (27.88% at 8 months, and 44.55% at 12 months). Of particular importance, dopaminergic cell death was found to be phenotype-dependent. Up to three distinct dopaminergic cell phenotypes were found, comprising (i) non-pigmented dopaminergic cells (TH+/NMel−), (ii) neuromelanized neurons (TH+/NMel+) and (iii) pigmented neurons that lost the TH phenotype (TH−/NMel+), the latter also known as “ghost cells” that are likely those that are more vulnerable to degeneration (Fig. 6C). Pigmented dopaminergic neurons (TH+/NMel+) were observed as early as one month post-viral administration (Figs. 2 and 6), although they represent a minimal fraction of the total number of dopaminergic neurons (3.12%). The number of TH+/NMel+ neurons increased at 4 months, when ghost cells were first observed. These two populations of pigmented neurons (TH+/NMel+ and TH−/NMel+) collectively accounted for 45.93% of the total number of dopaminergic neurons, meaning that by 4 months, roughly half of the SNpc neurons are pigmented. The ratio between pigmented and non-pigmented neurons increased at 8 and 12 months (74.57% at 8 months and 68.95% at 12 months). The most relevant decline in the number of TH+ neurons was observed from 1 to 4 months and from 4 to 8 months. Beyond 8 months, the number of TH+/NMel− neurons reached a plateau, likely meaning that non-pigmented neurons are more resilient to degeneration. The temporal dynamics for TH+/NMel+ and TH−/NMel+ neuronal phenotypes are roughly similar. These two cellular phenotypes reached a maximum peak at 8 months, followed by a slight decline at 12 months (Fig. 6, Supplementary Fig. 4).

Although all three different cellular phenotypes were also observed in the VTA, these neurons are far less vulnerable than those of the SNpc. Compared to the numbers of non-pigmented TH+ neurons, neuromelanized neurons (NMel+/TH+ & NMel+/TH−) barely represented half of the total dopaminergic cells in the VTA. Moreover, although a gradual cell loss was observed over time, such cellular neurodegeneration was smaller than in the SNpc (Fig. 6).

### Motor phenotype

**Fig. 7:**
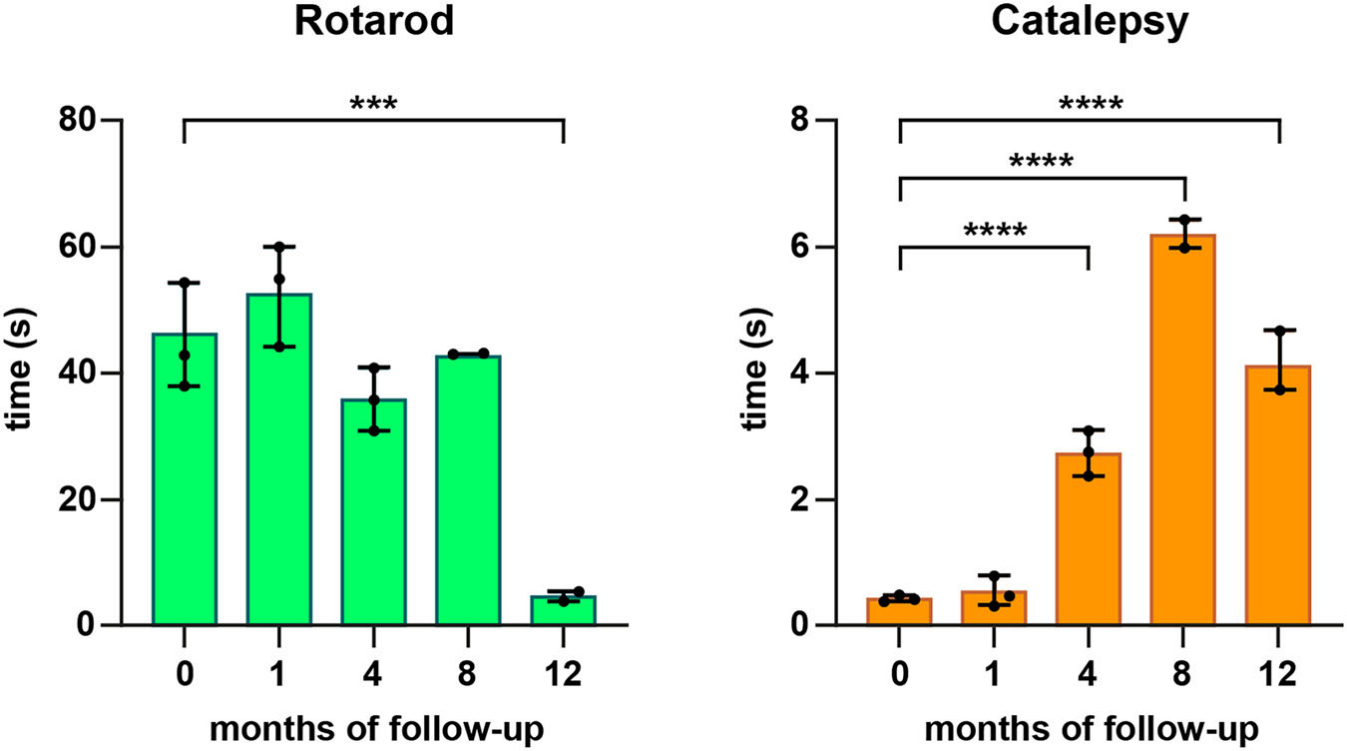
Motor readouts. The underlying nigrostriatal damage induced the appearance of a Parkinsonian-like motor impairment as observed with the conducted motor evaluation. Statistically significant differences were found with the rotarod test at 12 months of follow-up compared to controls (*p* < 0.0001), whereby the catalepsy test was more sensitive in addressing a motor readout. Data are represented as mean ± SEM, one-way ANOVA and Dunnett’s multiple comparison test, *p* < 0.001 was obtained for comparisons between control specimens and animals with 4, 8, and 12 months of survival times.

A time-dependent motor phenotype was observed in parallel to the extent of the nigrostriatal lesion. Compared to control animals, the experimental group comprising mice with a follow-up of 12 months exhibited a statistically significant difference in the rotarod test (Fig. 7). Moreover, the catalepsy test resulted in a higher sensitivity, where significant differences were found in all experimental groups beyond 4 months post-delivery of AAV9-P31-*hTyr* (Fig. 7). In summary and although each experimental group was made of a relatively low number of subjects, a clear time-dependent motor phenotype was observed with minimal inter-group differences.

### Peripheral organs

Since AAV9-P31-*hTyr* was delivered intravenously, samples from peripheral organs were taken at each time point to further address the potential presence of pigmentation. Analyses comprised paraffin-embedded tissue samples obtained from the lungs, heart, liver, spleen, small and large intestines, adrenal gland, kidney, striatal skeletal muscle, and gonads. Initial screenings were based on sections stained with H&E and non-stained sections. From all peripheral organs analyzed, high pigmentation levels were found in the red pulp of the spleen, beginning at 4 months post-viral delivery and gradually increasing over time (Supplementary Fig. 5A and B). Furthermore, a very weak NMel accumulation was found in the sarcoplasmic cones of the cardiomyocytes at 12 months post-injection of AAV9-P31-*hTyr* (Supplementary Fig. 5C and D). Pigmentation was never observed in peripheral organs other than the spleen and heart, irrespective of the follow-up time.

## Discussion

**Fig. 8:**
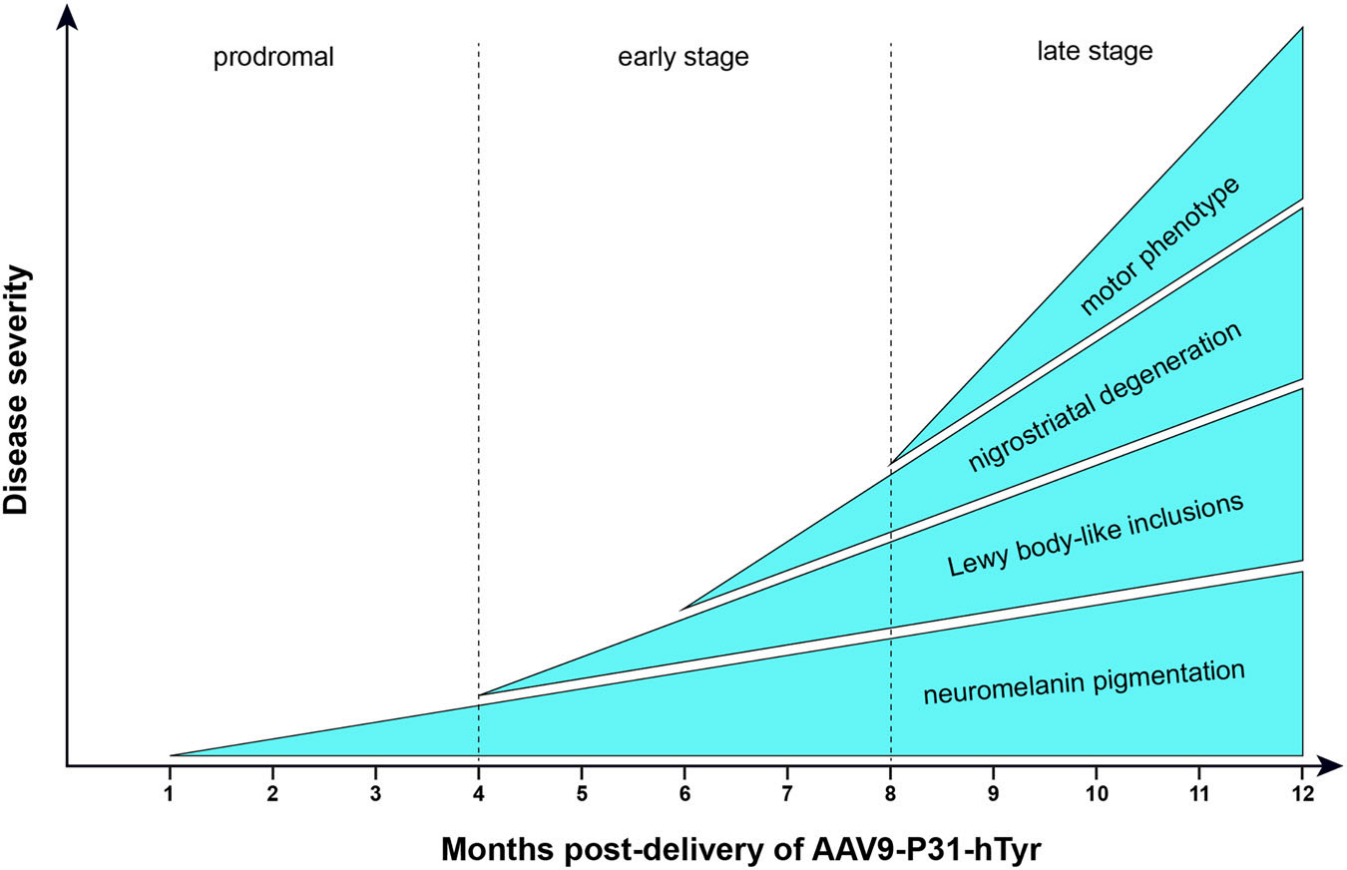
Outcomes of the PIGMO model. Schematic representation of the newly introduced PIGMO mouse model of Parkinson’s disease. The progressive cascade of events characterizing the PIGMO model defines well-characterized prodromal, early, and late stages, which would be instrumental for implementing novel disease-modifying therapeutic approaches. Since the model is progressive and has a wide therapeutic window, therapeutics can easily be administered once the underlying pathology has already started, and before reaching a non-returning point.

Here, a novel mouse model of PD has been developed and characterized, mimicking the known motor and pathological signatures typically observed in human PD. The intravenous administration of AAV9-P31-*hTyr* induced (i) a bilateral, ongoing pigmentation of catecholaminergic neurons in the SNpc, VTA, and LC, (ii) a time-dependent LBL pathology in neuromelanized neurons, (iii) a progressive nigrostriatal degeneration, and (iv) a PD-related motor phenotype (Fig. 8). Moreover, and in keeping with earlier animal models of enhanced neuromelanin pigmentation^9–11^, the obtained data supported that neuromelanin accumulation beyond a given threshold triggers an endogenous synucleinopathy in the form of LBLs.

The conducted study took advantage of a novel AAV9 capsid library^20^ designed by Voyager Therapeutics (Lexington, MA, USA) using the so-called TRACER platform (tropism redirection of AAV by cell-type-specific expression of mRNA). AAV9 capsid variants were engineered to bypass the BBB by targeting the carbonic anhydrase IV receptor, a protein expressed on the surface of endothelial cells^22^. Among the obtained capsid libraries, the P31 variant was chosen since it seems to be the best-performing one in terms of whole-brain transduction^20^. Upon intravenous administration, AAV9-P31 crossed the BBB and efficiently spread throughout the entire CNS, with a limited liver tropism. Neurons and astrocytes were transduced in the brain and spinal cord according to region-specific patterns^21^.

There is a continuous race for the development of AAV capsid variants with the ability to bypass the BBB, in an attempt to transduce the brain and spinal cord. Although AAV9 has some degree of BBB penetrance, the first capsid specifically designed for this purpose was AAV9-PHP.B, and a subsequent variant known as AAV9-PHP.eB^25^. These capsids bypass the BBB by targeting the LY6A cellular receptor^26,27^. Since its introduction, PHP.B and PHP.eB have been widely used to achieve a broad brain transduction following systemic deliveries. These viral vector capsids are specific for the C57BL/6J mouse strain, and cannot be used in other mouse strains, as well as in non-human primates^27–31^. Compared to PHP.B and PHP.eB, the AAV9-P31 capsid variant exhibited similar performance across different mouse strains tested so far, such as C57BL/6J, Balb/c, and FVB/N^21,32^. Moreover, although the C57BL/6J strain is widely used in neuroscience research, it carries a spontaneous deletion of the endogenous SNCA gene, resulting in the lack of expression of alpha-synuclein. This genetic deficiency prevented us from using this strain for the development of the PIGMO model. Conversely, FVB/N mice express endogenous alpha-synuclein, and this strain has the genetic background of a humanized alpha-synuclein mouse model.

Although the potential link between NMel and dopaminergic cell vulnerability has long been postulated^12–14^, this association was often neglected since rodents, as the most commonly used laboratory experimental animals, completely lack NMel pigmentation in SNpc, VTA, and LC neurons. Furthermore, NMel pigmentation of aged dopaminergic cells parallels the accumulation of toxic chemical compounds such as metallic ions that exhibit a high binding affinity with melanins^33,34^. Furthermore, the chelating nature of NMel allows interaction with neurotoxicants like MPP+ and alpha-synuclein^35,36^. In parallel, accumulated clinical evidence supported a bidirectional link between the incidence of Parkinson’s disease and melanoma^15^. Interestingly, alpha-synuclein expression in malignant melanocytes was recently reported elsewhere^16^.

Existing evidence paved the way for the implementation of novel animal models of PD based on the AAV-mediated enhanced expression of human tyrosinase aimed to induce NMel accumulation in dopaminergic neurons. These approaches were first tested in rats through the intranigral delivery of AAV1-*hTyr*^9^ and later upgraded to non-human primates^10^. Both strategies lead to the characterization of animal models mimicking the known neuropathological hallmarks of PD with unprecedented accuracy, comprising a time-dependent pigmentation of SNpc neurons, presence of intracytoplasmic LBL aggregates, and a progressive dopaminergic cell death. Moreover, rats injected with AAV1-*hTyr* displayed an evident motor phenotype^9^, whereby a circuit-specific anterograde spread of alpha-synuclein towards the cerebral cortex has been observed in macaques^10^. Although the mechanisms underlying the cross-talk between NMel and alpha-synuclein have not yet been elucidated in detail, these models provided the first demonstration linking NMel accumulation above a given threshold as the key factor leading to the development of an endogenous synucleinopathy in the form of LBLs. In this regard, gene therapy strategies designed to reduce NMel levels have succeeded in preventing the subsequent synucleinopathy and thus leading to dopaminergic cell neuroprotection^9,37^. Moreover, the recent introduction of a tissue-specific transgenic mouse model based on the constitutive expression of the *hTyr* gene under the control of a tyrosine hydroxylase promoter (thus leading to a restricted expression in catecholaminergic neurons throughout the entire mouse brain) represents another step forward in the same direction^11^. The transgenic *hTyr* mouse line can be regarded as a feasible alternative to the PIGMO model (or vice versa).

Available animal models of PD have been instrumental in setting up the currently available therapeutic arsenal and obtaining a deeper understanding of the mechanisms underlying the pathophysiological events that typically characterize this neurodegenerative disorder. By definition, each model serves specific research goals, meaning that not a single model fully recapitulates the inherent clinicopathological complexity of PD, and therefore can hardly predict the therapeutic efficacy of novel candidates, such as those with a potential disease-modifying effect^1,4,6^.

Besides neurotoxin-based animal models, at present the most popular mouse models can be broadly categorized into those related to alpha-synuclein^38–41^ and those focused on highly penetrant rare genetic mutations linked to familial forms of PD, such as those related to the PARK family, LRRK2, PINK1, GBA1, and DJ-1^5,42,43^. Mouse models outside these categories are the Mitopark mouse^44^, as well as those being recently introduced, such as the one based on loss of functional mitochondrial complex I^45^ and the transgenic mouse line M12^46^. In general terms, and regardless of the chosen rodent model of PD, not a single model managed to mimic the real human disorder; however, these models have a proven record of success when investigating specific disease mechanisms. For instance, viral enhancement of alpha-synuclein expression in the SNpc often resulted in a non-consistent dopaminergic degeneration^47^.

Accordingly, for most of the mouse models listed above, the PIGMO model introduced here can be regarded as complementary instead of an alternative model. In other words, the intravenous delivery of AAV9-P31-*hTyr* can easily be used as a feasible strategy enabling a second insult intended to boost the PD phenotype in models lacking the right outcomes.

The PIGMO model introduced here has several advantages, such as (i) simplicity of implementation by merely needing a systemic administration of the BBB-penetrant viral vector encoding the *hTyr* gene leading to a bilateral PD model without the need to perform stereotaxic surgeries for intraparenchymal injections, (ii) a wide therapeutic window enabling testing disease-modifying therapeutic interventions, and (iii) a consistent pattern of neuropathological and motor readouts, enabling administration of therapeutics once the neurodegenerative processes are already ongoing but before reaching a non-returning point.

## Methods

### Study design

This study aims to develop a novel pigmented mouse model of PD (PIGMO) that would enhance existing knowledge of underlying mechanisms leading to dopaminergic neurodegeneration, further increasing the chances of success when testing disease-modifying therapeutic candidates. The goal was to generate an accessible and progressive mouse model, without the inherent biases of stereotaxic surgeries, that mimics the neuropathological signatures of this disorder to the best possible extent, together with the characteristic progressive motor phenotype. Accordingly, we sought to evaluate whether the systemic delivery of a BBB-penetrant AAV9 capsid variant coding for the human tyrosinase gene (AAV9-P31-*hTyr*) can induce a bilateral, time-dependent pigmentation of catecholaminergic brain centers related to PD pathophysiology, such as the substantia nigra pars compacta (SNpc), ventral tegmental area (VTA), and locus coeruleus (LC). In keeping with earlier data obtained upon intranigral deliveries of AAV1-*hTyr*^9,10^, Lewy body-like pathology and progressive cell death were expected to be found in catecholaminergic neurons of the CNS (in particular, within dopaminergic neurons of the SNpc). Four experimental groups of adult mice were injected in the retro-ocular vein plexus with AAV9-P31-*hTyr* or an inactivated construct for control purposes (AAV9-P31-stop-*hTyr*). Each experimental group was made up of four animals, comprising one control subject injected with AAV9-P31-stop-*hTyr* and three mice injected with AAV9-P31-*hTyr*. To delineate a timeline, experimental groups 1–4 were euthanized at 1, 4, 8, and 12 months post-viral deliveries, and motor behavior was assessed with rotarod and catalepsy tests before sacrifice. Brain tissue samples were processed for histological analysis, comprising intracellular neuromelanin levels, intracytoplasmic inclusions, and nigrostriatal damage.

### Experimental animals

Adult male and female mice (FVN/N strain; Charles River Laboratories, Barcelona, Spain) were used in this study. Animal handling and experimental procedures were conducted in accordance with the European Council Directive 2010/63/EU and in keeping with the Spanish legislation (RD 53/2013). The experimental design was approved by the Ethical Committee for Animal Experimentation of the University of Navarra (ref. CEEA 110-21) as well as by the Department of Animal Welfare of the Government of Navarra.

### Viral vector production

Recombinant AAV vector serotype 2/9 capsid variant P31^20^ expressing the human tyrosinase cDNA driven by the CMV promoter (AAV9-P31-*hTyr*) and the corresponding control vector with an inactivated human tyrosinase cDNA sequence with a stop codon (AAV9-P31-stop-*hTyr*) were produced at the Viral Vector Core Production Unit of the University Autónoma of Barcelona, Spain (UPV-UAB; https://www.viralvector.eu/). In brief, AAVs were produced by triple transfection of 2 × 10^8^ HEK293 cells with 250 μg pAAV, 250 μg pRepCap, and 500 μg pXX6 plasmid mixed with polyethylenimine (Sigma-Aldrich). The UPV-UAB core facility generated a pAAV plasmid containing the inverted terminal repeats (ITRs) of the AAV2 genome, a multi-cloning site to facilitate cloning of expression cassettes, and an ampicillin resistance gene for selection. Two days after transfection, cells were harvested by centrifugation, resuspended in 30 mL of 20 mM NaCl, 2 mM MgCl_2_, and 50 mM Tris–HCl (pH 8.5), and lysed by three freeze-thawing cycles. Cell lysate was clarified by centrifugation, and the AAV particles were purified from the supernatant by iodixanol gradient as previously described^48^. Next, the clarified lysate was treated with 50 U/mL of benzonase (Novagen; 1 h at 37 °C) and centrifuged. The vector-containing supernatant was collected and adjusted to 200 mM NaCl using a 5 M stock solution. To precipitate the virus from the clarified cell lysate, polyethylene glycol (Sigma-Aldrich) was added to a final concentration of 8%, and the mixture was incubated (3 h, 4 °C) and centrifuged. Pellets containing AAVs were resuspended in 20 mM NaCl, 2 mM MgCl_2_, and 50 mM Tris–HCl (pH 8.5) and incubated for 48 h at 4 °C. The AAV titration method used was based on the quantitation of encapsulated DNA with the fluorescent dye PicoGreen®. Obtained vector concentrations were 1.16 × 10^13^ GC/mL for AAV9-P31-*hTyr* and 1.76 × 10^13^ GC/mL for AAV9-P31-stop-*hTyr*. The plasmid maps and sequences are provided in Supplementary Fig. 1.

### Systemic deliveries of AAVs

Animals were anesthetized with isoflurane and treated with the analgesic buprenorphine (Brupex®; 0.1 mg/kg). A drop of ophthalmic anesthetic (0.5% proparacaine hydrochloride) was also placed on the eye receiving the injection. Viral vectors were administered systemically through a retro-orbital injection of the venous sinus^49^, a more humane approach than alternative intravascular deliveries, and commonly used as the standard choice for viral vector deliveries in mice^25,50–58^. Delivery was performed in the right retro-orbital sinus (right-handed operator) with a 30-gauge, 0.5-in insulin needle and syringe (Omnican®; ref. 9151117S; Braun, Melsungen, Germany), comprising a total volume of 100 μL made of 50 μL of AAVs diluted in 50 μL of PBS, resulting in a final viral dose of 2.9 × 10^11^ GC/animal for AAV9-P31-*hTyr* and 4.4 × 10^11^ GC/animal for AAV9-P31-stop-*hTyr*. The AAV suspension was slowly and smoothly injected. Once the injection was completed, the needle was slowly withdrawn to minimize reflux through the injection site. Experimental subjects were divided into four groups, each made of one control specimen and three treated animals.

### Necropsy and tissue processing

Animals were anesthetized with ketamine/xylazine (80/10 mg/kg) and perfused transcardially with a saline solution (0.9% NaCl; 5 min; 9.5 mL/min), followed by 25 mL of a 4% paraformaldehyde-buffered solution (PFA). Once perfusion was completed, the brains were removed from the skull and post-fixed in 4% PFA for 24 h and then stored in a cryoprotectant solution containing 20% glycerin and 2% DMSO in neutral PBS. Next, frozen coronal sections (40 μm-thick) were obtained in a sliding microtome (Microm HM-400) and collected in 0.125 M PBS pH 7.4 as 10 series of adjacent sections. These series were used for (i) direct neuromelanin visualization; (ii) immunoperoxidase detection of TH; (iii) Masson-Fontana stain; (iv) cytoarchitectural stain with Neutral Red; (v) proteinase K-pretreated, triple immunofluorescent detection of alpha-synuclein (α-Syn), P62, and tyrosine hydroxylase (TH), combined with brightfield visualization of neuromelanin (NMel); (vi) proteinase K-pretreated, triple immunofluorescent detection of phosphorylated α-Syn(P-Ser 129), P62, and TH, combined with brightfield visualization of NMel; and (vii) proteinase K-pretreated, triple immunofluorescent detection of phosphorylated α-Syn(P-Ser 129), P62, and ubiquitin, combined with brightfield visualization of NMel. The remaining series of sections was stored at −80 °C until further use, if required. A detailed protocol for necropsy and tissue processing is available elsewhere^32,59^.

A complete list of the used primary and bridge antisera (biotinylated or Alexa® dye-conjugated) is provided in Table 1.

**Table 1:** List of antibodies and reagents

Antibody	Dilution	Incubation time	Source	Identifier	RRID
Goat anti-tyrosine hydroxylase (polyclonal)	1:500	Overnight	Abcam	Cat# ab101853	AB_10710873
Mouse anti-α-synuclein (monoclonal)	1:40	Overnight	Leica	Cat# NCL-L-ASYN	AB_442103
Mouse anti-α-synuclein P-Ser129 (monoclonal)	1:1000	Overnight	Fujifilm Wako	Cat#015-25191	AB_2537218
Guinea Pig anti-P62 (polyclonal)	1.1000	Overnight	PROGEN	Cat# GP62-C	AB_2687531
Mouse anti-ubiquitin (monoclonal)	1:500	Overnight	Abcam	Cat# ab7254	AB_305802
Donkey anti-Goat (Biotin-SP)	1:600	120 min	Jackson	Cat# 705-065-147	AB_2340397
Donkey anti-Goat (Alexa488)	1.200	120 min	Molecular Probes	Cat# A-11055	AB_2534102
Donkey anti-Goat (Alexa350)	1.200	120 min	Molecular Probes	Cat# A-21081	AB_141521
Donkey anti-Goat (Alexa 633)	1:200	120 min	Molecular Probes	Cat# A-21082	AB_141493
Donkey anti-Guinea Pig (Alexa594)	1:200	120 min	Jackson	Cat# 706-585-148	
Donkey anti-Mouse (Alexa488)	1:200	120 min	Molecular Probes	Cat# A-21202	AB_141607
Donkey anti-Mouse (Alexa546)	1:200	120 min	Thermo Fisher	Cat# A-10036	AB_11180613
Donkey anti-Rabbit (Alexa488)	1:200	120 min	Molecular Probes	Cat# A-21206	AB_2535792

Commercial assays	Dilution	Incubation time	Source	Identifier
ABC Kit Standard		60 min	Vector Labs	Cat# PK-4000
Neutral Red	0.40%	1 min	Sigma	Cat# 72210
Peroxidase chromogen V-VIP		1 min	Vector Labs	Cat# SK-4600
Proteinase K	1 mg/mL	10 min	Invitrogen	Cat# 25530049

### Analysis of pigmented neurons

Every 10th section was counterstained with Neutral Red and used to estimate the number of pigmented vs. non-pigmented neurons in the SNpc and VTA. Quantification was carried out through a dedicated, deep-learning bi-layered algorithm prepared with Aiforia^60^ (www.aiforia.com) according to the available protocol (10.17504/protocols.io.bp2l6xdwrlqe/v1). Five equally spaced coronal sections comprising the whole rostrocaudal extent of the SNpc and the VTA were sampled per animal. Sections were scanned at ×20 in an Aperio CS2 scanner (Leica) and uploaded to the Aiforia cloud. The boundaries of the SNpc and VTA were outlined at low magnification (taking the exit of the 6th cranial nerve as reference), and the algorithm was then used for quantifying the desired neuronal populations.

Furthermore, the location of three distinct neuronal profiles in the SNpc and in the VTA (TH+/NMel+; TH+/NMel− and TH−/NMel+) was carried out by camera lucida drawings made from three equally spaced coronal sections at the level of the SNpc and VTA (comprising rostral, middle, and caudal midbrain levels) in each animal and for each time point.

### Quantification of intracellular neuromelanin levels

The intracellular density of NMel pigmentation was analyzed by measuring optical densitometry at the single-cell level with Fiji ImageJ software (NIH, USA). Obtained values were converted to a logarithmic scale according to the available protocol^61^. The number of analyzed neurons within each region of interest (ROI) and at each time point is summarized in Table 2.

**Table 2:** Number of neurons used for quantifying intracellular neuromelanin levels

Time post-injection	NMel+ neurons (SNpc)	NMel+ neurons (VTA)	NMel+ neurons (LC)	Total NMel+ neurons
1 month	Mouse #1: 83 Mouse #2: 57 Mouse #3: 49	Mouse #1: 8 Mouse #2: 9 Mouse #3: 8	Mouse #1: 0 Mouse #2: 0 Mouse #3: 0	214
4 months	Mouse #4: 206 Mouse #5: 236 Mouse #6: 167	Mouse #4: 45 Mouse #5: 17 Mouse #6: 106	Mouse #4: 0 Mouse #5: 0 Mouse #6: 9	786
8 months	Mouse #7: 194 Mouse #8: 187 Mouse #9: 125	Mouse #7: 113 Mouse #8: 99 Mouse #9: 79	Mouse #7: 10 Mouse #8: 39 Mouse #9: 23	869
12 months	Mouse #10: 164 Mouse #11: 186 Mouse #12: 129	Mouse #10: 114 Mouse #11: 12 Mouse #12: 71	Mouse #10: 25 Mouse #11: 18 Mouse #12: 55	774
Total NMel+ neurons	1783	681	179	2643

### Assessment of nigrostriatal damage

The second series of coronal sections was used for the immunohistochemical detection of TH (10.17504/protocols.io.8epv5x4wdg1b/v1). Sections rostral to the subthalamic nucleus (STN) were used for visualization of TH+ axon terminals at the level of the striatum using a standard diaminobenzidine peroxidase substrate (DAB), resulting in a dark brown precipitate. For each animal, TH optical intensities were measured in six equally spaced coronal sections of the striatum (pre- and post-commissural levels) with Fiji ImageJ software (NIH, USA), and the obtained values were converted to a logarithmic scale^61^. Coronal sections caudal to the STN were used for the immunohistochemical detection of TH with purple peroxidase substrate (V-VIP^62,63^). The V-VIP chromogen was chosen for TH detection in the SNpc, VTA, and LC since a better contrast is obtained for the NMel pigmentation against a purple background rather than the dark brown color that characterizes the DAB peroxidase substrate. The number of TH+ neurons in the SNpc and the VTA was quantified across six equally spaced coronal sections covering the whole rostrocaudal extent of the mesencephalon with a dedicated Aiforia® algorithm (10.17504/protocols.io.36wgq3x55lk5/v1).

### Neuronal inclusions

The presence of intracytoplasmic inclusions in pigmented catecholaminergic neurons of the SNpc, VTA, and LC was assessed under the confocal microscope by triple immunofluorescent detection of TH combined with characteristic markers of Lewy body pathology, such as P62 and phosphorylated alpha-synuclein (P-Ser129). One additional brightfield channel was included to visualize NMel pigmentation. Pre-digestion with proteinase K was conducted before immunofluorescent staining to remove soluble forms of alpha-synuclein. Moreover, another series of sections was used for triple immunofluorescent detection of phosphorylated alpha-synuclein, P62, and ubiquitin, to demonstrate that intracytoplasmic inclusions are ubiquitinated^10^.

### Evaluation of motor phenotype

Standardized behavioral tests (rotarod and catalepsy tests^32,59^) were used to evaluate motor readouts at different time-points post-administration of AAV9-P31-*hTyr* and AAV9-P31-stop-*hTyr* (1, 4, 8, and 12 months). In brief, for the rotarod test, mice were placed on a revolving bar accelerating from 4 to 40 rpm over 5 min. To acclimate, animals were exposed for 30 s on day 1 and one minute on day 2. On the test day, mice underwent two trials with a 30-min interval, and results were expressed as the average latency to fall. The catalepsy test was used to measure muscular rigidity by placing the forepaws of the experimental subjects on a 4 cm high bar. The time taken to adjust the posture was recorded across three trials, with a 1-min resting interval.

### Statistical analyses

Statistical analyses were performed in GraphPad Prism version 9.0.2 for Windows and Stata 14 (Stata Corp. 2017, Stata Statistical Software Release 15, College Station, TX; StataCorp LLC). Relevant tests are listed in the figure legends. Species with *p* < 0.05 were considered statistically significant.

## Supplementary information

Suppl Figures 1-6

## Data Availability

Further information and requests for resources and reagents should be directed to and will be fulfilled by the corresponding author, Jose L. Lanciego (jlanciego@unav.es). The data, code, protocols, and key lab materials used and generated in this study are listed in a Key Resource Table alongside their persistent identifiers at 10.5281/zenodo.15386050. No code was generated for this study; all data cleaning, preprocessing, analysis, and visualization were performed using Fiji ImageJ, GraphPad Prism 9.0.2, Stata 14, and Aiforia.
