# Supplementary material for: Introducing PIGMO, a novel PIGmented MOuse model of Parkinson’s disease
Source: NPJ Parkinsons Dis. 2026 Feb 11;12:72. doi: 10.1038/s41531-026-01289-9 (PMC13005038; doi:10.1038/s41531-026-01289-9)
Supplement: Supplementary file 1 — Suppl Figures 1-6 [file 41531_2026_1289_MOESM1_ESM.pdf]

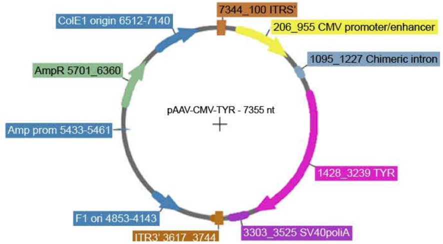

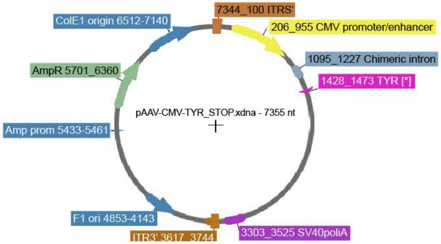

pAAV-CMV-TYR sequence

CAGCAGCTGCGCGCTCGCTCGCTCACTGAGGCCGCCCGGGCAAAGCCCGGGCGTCGGGCGACCTTTGGTCGCCCGGCCTCAGTGAGCGAGCGAGCGCG
CAGAGAGGGAGTGGCCAACTCCATCACTAGGGGTTCCTTGTAGTTAATGATTAACCCGCCATGCTACTTATCTACGTAGCCATGCTCTAGACATGGCTCGAC
AGATCTCAATATTGGCCATTAGCCATATTATTCATTGGTTATATAGCATAAATCAATATTGGCTATTGGCCATTGCATACGTTGTATCTATATCATAATATGTAC
ATTTATATTGGCTCATGTCCAATATGACCGCCATGTTGGCATTGATTATTGACTAGTTATTAATAGTAATCAATTACGGGGTCATTAGTTCATAGCCCATATAT
GGAGTTCCGCGTTACATAACTTACGGTAAATGGCCCGCCTGGCTGACCGCCCAACGACCCCCGCCCATTGACGTCAATAATGACGTATGTTCCCATAGTAA
CGCCAATAGGGACTTTCCATTGACGTCAATGGGTGGAGTATTTACGGTAAACTGCCCACTTGGCAGTACATCAAGTGTATCATATGCCAAGTCCGCCCCCTA
TTGACGTCAATGACGGTAAATGGCCCGCCTGGCATTATGCCCAGTACATGACCTTACGGGACTTTCCTACTTGGCAGTACATCTACGTATTAGTCATCGCTA
TTACCATGGTGATGCGGTTTTGGCAGTACACCAATGGGCGTGGATAGCGGTTTGACTCACGGGGATTTCCAAGTCTCCACCCCATTGACGTCAATGGGAGT
TTGTTTTGGCACCAAAATCAACGGGACTTTCCAAAATGTCGTAACAACTGCGATCGCCCGCCCCGTTGACGCAAATGGGCGGTAGGCGTGTACGGTGGGAG
GTCTATATAAGCAGAGCTCGTTTAGTGAACCGTCAGATCACTAGAAGCTTTATTGCGGTAGTTTATCACAGTTAAATTGCTAACGCAGTCAGTGCTTCTGACA
CAACAGTCTCGAACTTAAGCTGCAGTGACTCTCTTAAGGTAGCCTTGCAGAAGTTGGTCGTGAGGCACTGGGCAGGTAAGTATCAAGGTTACAAGACAGGT
TTAAGGAGACCAATAGAAACTGGGCTTGTCGAGACAGAGAAGACTCTTGCGTTTCTGATAGGCACCTATTGGTCTTACTGACATCCACTTTGCCTTTCTCTCC
ACAGGTGTCCACTCCCAGTTCAATTACAGCTCTTAAGGCTAGAGTACTTAATACGACTCACTATAGGCTAGCCTCGACCTCGAGACGCGTGATAAACTTAAG
CTTGGTACCGAGCTCGGATCCACTAGTCCAGTGTGGTGGAATTCTGCAGATATCCAGCACAGTGGCGGCCGCTCGACCGACCTTGTGAGGACTAGAGGAA
GAATGCTCCTGGCTGTTTTGTACTGCCTGCTGTGGAGTTTCCAGACCTCCGCTGGCCATTTCCCTAGAGCCTGTGTCTCCTCTAAGAACCTGATGGAGAAG
GAATGCTGTCCACCGTGGAGCGGGGACAGGAGTCCCTGTGGCCAGCTTTCAGGCAGAGGTTCCTGTCAGAATATCCTTCTGTCCAATGCACCACTTGGGC
CTCAATTTCCCTTCACAGGGGTGGATGACCGGGAGTCGTGGCCTTCCGTCTTTTATAATAGGACCTGCCAGTGCTCTGGCAACTTCATGGGATTCAACTGTG
GAAACTGCAAGTTTGGCTTTTGGGGACCAAACTGCACAGAGAGACGACTCTTGGTGAGAAGAAACATCTTCGATTTGAGTGCCCCAGAGAAGGACAAATTTT
TTGCCTACCTCACTTTAGCAAAGCATACCATCAGCTCAGACTATGTCATCCCCATAGGGACCTATGGCCAAATGAAAAATGGATCAACACCCATGTTTAACGA
CATCAATATTTATGACCTCTTTGTCTGGATGCATTATTATGTGTCAATGGATGCACTGCTTGGGGGATCTGAAATCTGGAGAGACATTGATTTTGCCCATGAA
GCACCAGCTTTTCTGCCTTGGCATAGACTCTTCTTGTTGCGGTGGGAACAAGAAATCCAGAAGCTGACAGGAGATGAAAACTTCACTATTCCATATTGGGAC
TGGCGGGATGCAGAAAAGTGTGACATTTGCACAGATGAGTACATGGGAGGTCAGCACCCCACAAATCCTAACTTACTCAGCCCAGCATCATTCTTCTCCTCT
TGGCAGATTGTCTGTAGCCGATTGGAGGAGTACAACAGCCATCAGTCTTTATGCAATGGAACGCCCGAGGGACCTTTACGGCGTAATCCTGGAAACCATGA
CAAATCCAGAACCCCAAGGCTCCCCTCTTCAGCTGATGTAGAATTTTGCCTGAGTTTGACCCAATATGAATCTGGTTCCATGGATAAAGCTGCCAATTTCAGC
TTTAGAAATACACTGGAAGGATTTGCTAGTCCACTTACTGGGATAGCGGATGCCTCTCAAAGCAGCATGCACAATGCCTTGCACATCTATATGAATGGAACA
ATGTCCCAGGTACAGGGATCTGCCAACGATCCTATCTTCCTTCTTCACCATGCATTTGTTGACAGTATTTTTGAGCAGTGGCTCCGAAGGCACCGTCCTCTT
CAAGAAGTTTATCCAGAAGCCAATGCACCCATTGGACATAACCGGGAATCCTACATGGTTCCTTTTATACCACTGTACAGAAATGGTGATTTCTTTATTTCATC
CAAAGATCTGGGCTATGACTATAGCTATCTACAAGATTCAGACCCAGACTCTTTTCAAGACTACATTAAGTCCTATTTGGAACAAGCGAGTCGGATCTGGTCA
TGGCTCCTTGGGGCGGCGATGGTAGGGGCCGTCCTCACTGCCCTGCTGGCAGGGCTTGTGAGCTTGCTGTGTCGTCACAAGAGAAAGCAGCTTCCTGAAG
AAAAGCAGCCACTCCTCATGGAGAAAGAGGATTACCACAGCTTGTATCAGAGCCATTTATAAAAGGCTTAGGCAATAGAGTAGGGCCAAAAAGCCTGACCTC
ACTCTAACTCAAAGTAATGTCCAGGTTCCCAGAGAATATCTGCTGGTATTTTTCTGTAAAGACCATTTGCAAAATTGTAACCTAATACAAAGTGTAGCCTTCTT
CCAACTCAGGTAGAACACACCTGTCTTTGTCTTGCTGTTTTCACTCAGCCCTTTTAACATTTTCCCCTAAGCCCCTAGAGGGCCCGTTTATCGGATCCCGGC
CGGCGGCCGCTTCCCTTTAGTGAGGGTTAATGCTTCGAGCAGACATGATAAGATACATTGATGAGTTTGGACAAACCACAACTAGAATGCAGTGAAAAAAAT
GCTTTATTTGTGAAATTTGTGATGCTATTGCTTTATTTGTAACCATTATAAGCTGCAATAAACAAGTTAACAACAACAATTGCATTCATTTTATGTTTCAGGTTCA
GGGGGAGATGTGGGAGGTTTTTTAAAGCAAGTAAAACCTCTACAAATGTGGTAAAATCCGATAAGGACTAGAGCATGGCTACGTAGATAAGTAGCATGGC
GGGTTAATCATTAACTACAAGGAACCCCTAGTGATGGAGTTGGCCACTCCCTCTCTGCGCGCTCGCTCGCTCACTGAGGCCGGGCGACCAAAGGTCGCCC
GACGCCCGGGCTTTGCCCGGGCGGCCTCAGTGAGCGAGCGAGCGCGCCAGCTGGCGTAATAGCGAAGAGGCCCGCACCGATCGCCCTTCCCAACAGTT
GCGCAGCCTGAATGGCGAATGGAATTCCAGACGATTGAGCGTCAAAATGTAGGTATTTCCATGAGCGTTTTTCCGTTGCAATGGCTGGCGGTAATATTGTTC
TGGATATTACCAGCAAGGCCGATAGTTTGAGTTCTTCTACTCAGGCAAGTGATGTTATTACTAATCAAAGAAGTATTGCGACAACGGTTAATTTGCGTGATGG
ACAGACTCTTTTACTCGGTGGCCTCACTGATTATAAAAACACTTCTCAGGATTCTGGCGTACCGTTCCTGTCTAAAATCCCTTTAATCGGCCTCCTGTTTAGC
TCCCGCTCTGATTCTAACGAGGAAAGCACGTTATACGTGCTCGTCAAAGCAACCATAGTACGCGCCCTGTAGCGGCGCATTAAGCGCGGCGGGTGTGGTG
GTTACGCGCAGCGTGACCGCTACACTTGCCAGCGCCCTAGCGCCCGCTCCTTTCGCTTTCTTCCCTTCCTTTCTCGCCACGTTCGCCGGCTTTCCCCGTCA
AGCTCTAAATCGGGGGCTCCCTTTAGGGTTCCGATTTAGTGCTTTACGGCACCTCGACCCCAAAAAACTTGATTAGGGTGATGGTTCACGTAGTGGGCCATC
GCCCTGATAGACGGTTTTTCGCCCTTTGACGTTGGAGTCCACGTTCTTTAATAGTGGACTCTTGTTCCAAACTGGAACAACACTCAACCCTATCTCGGTCTAT
TCTTTTGATTTATAAGGGATTTTGCCGATTTCGGCCTATTGGTTAAAAAATGAGCTGATTTAACAAAAATTTAACGCGAATTTTAACAAAATATTAACGTCTACA
ATTTAAATATTTGCTTATACAATCTTCCTGTTTTTGGGGCTTTTCTGATTATCAACCGGGGTACATATGATTGACATGCTAGTTTTACGATTACCGTTCATCGAT
TCTCTTGTTTGCTCCAGACTCTCAGGCAATGACCTGATAGCCTTTGTAGAGACCTCTCAAAAATAGCTACCCTCTCCGGCATGAATTTATCAGCTAGAACGGT
TGAATATCATATTGATGGTGATTTGACTGTCTCCGGCCTTTCTCACCCGTTTGAATCTTTACCTACACATTACTCAGGCATTGCATTTAAAATATATGAGGGTT
CTAAAAATTTTTATCCTTGCGTTGAAATAAAGGCTTCTCCCGCAAAAGTATTACAGGGTCATAATGTTTTTGGTACAACCGATTTAGCTTTATGCTCTGAGGCT
TTATTGCTTAATTTTGCTAATTCTTTGCCTTGCCTGTATGATTTATTGGATGTTGGAATCGCCTGATGCGGTATTTTCTCCTTACGCATCTGTGCGGTATTTCAC
ACCGCATATGGTGCACTCTCAGTACAATCTGCTCTGATGCCGCATAGTTAAGCCAGCCCCGACACCCGCCAACACCCGCTGACGCGCCCTGACGGGCTTG
TCTGCTCCCGGCATCCGCTTACAGACAAGCTGTGACCGTCTCCGGGAGCTGCATGTGTCAGAGGTTTTCACCGTCATCACCGAAACGCGCGAGACGAAAG
GGCCTCGTGATACGCCTATTTTTATAGGTTAATGTCATGATAATAATGGTTTCTTAGACGTCAGGTGGCACTTTTCGGGGAAATGTGCGCGGAACCCCTATTT
GTTTATTTTTCTAAATACATTCAAATATGTATCCGCTCATGAGACAATAACCCTGATAAATGCTTCAATAATATTGAAAAAGGAAGAGTATGAGTATTCAACATT
TCCGTGTCGCCCTTATTCCCTTTTTTGCGGCATTTTGCCTTCCTGTTTTTGCTCACCCAGAAACGCTGGTGAAAGTAAAAGATGCTGAAGATCAGTTGGGTGC
ACGAGTGGGTTACATCGAACTGGATCTCAACAGCGGTAAGATCCTTGAGAGTTTTCGCCCCGAAGAACGTTTTCCAATGATGAGCACTTTTAAAGTTCTGCT
ATGTGGCGCGGTATTATCCCGTATTGACGCCGGGCAAGAGCAACTCGGTCGCCGCATACACTATTCTCAGAATGACTTGGTTGAGTACTCACCAGTCACAG
AAAAGCATCTTACGGATGGCATGACAGTAAGAGAATTATGCAGTGCTGCCATAACCATGAGTGATAACACTGCGGCCAACTTACTTCTGACAACGATCGGAG
GACCGAAGGAGCTAACCGCTTTTTTGCACAACATGGGGGATCATGTAACTCGCCTTGATCGTTGGGAACCGGAGCTGAATGAAGCCATACCAAACGACGAG
CGTGACACCACGATGCCTGTAGCAATGGCAACAACGTTGCGCAAACTATTAACTGGCGAACTACTTACTCTAGCTTCCCGGCAACAATTAATAGACTGGATG
GAGGCGGATAAAGTTGCAGGACCACTTCTGCGCTCGGCCCTTCCGGCTGGCTGGTTTATTGCTGATAAATCTGGAGCCGGTGAGCGTGGGTCTCGCGGTA
TCATTGCAGCACTGGGGCCAGATGGTAAGCCCTCCCGTATCGTAGTTATCTACACGACGGGGAGTCAGGCAACTATGGATGAACGAAATAGACAGATCGCT
GAGATAGGTGCCTCACTGATTAAGCATTGGTAACTGTCAGACCAAGTTTACTCATATATACTTTAGATTGATTTAAAACTTCATTTTTAATTTAAAAGGATCTAG
GTGAAGATCCTTTTTGATAATCTCATGACCAAAATCCCTTAACGTGAGTTTTCGTTCCACTGAGCGTCAGACCCCGTAGAAAAGATCAAAGGATCTTCTTGAG
ATCCTTTTTTTCTGCGCGTAATCTGCTGCTTGCAAACAAAAAAACCACCGCTACCAGCGGTGGTTTGTTTGCCGGATCAAGAGCTACCAACTCTTTTTCCGAA
GGTAACTGGCTTCAGCAGAGCGCAGATACCAAATACTGTCCTTCTAGTGTAGCCGTAGTTAGGCCACCACTTCAAGAACTCTGTAGCACCGCCTACATACCT
CGCTCTGCTAATCCTGTTACCAGTGGCTGCTGCCAGTGGCGATAAGTCGTGTCTTACCGGGTTGGACTCAAGACGATAGTTACCGGATAAGGCGCAGCGGT
CGGGCTGAACGGGGGGTTCGTGCACACAGCCCAGCTTGGAGCGAACGACCTACACCGAACTGAGATACCTACAGCGTGAGCTATGAGAAAGCGCCACGC
TTCCCGAAGGGAGAAAGGCGGACAGGTATCCGGTAAGCGGCAGGGTCGGAACAGGAGAGCGCACGAGGGAGCTTCCAGGGGGAAACGCCTGGTATCTTT
ATAGTCCTGTCGGGTTTCGCCACCTCTGACTTGAGCGTCGATTTTTGTGATGCTCGTCAGGGGGGCGGAGCCTATGGAAAAACGCCAGCAACGCGGCCTTT
TTACGGTTCCTGGCCTTTTGCTGGCCTTTTGCTCACATGTTCTTTCCTGCGTTATCCCCTGATTCTGTGGATAACCGTATTACCGCCTTTGAGTGAGCTGATA
CCGCTCGCCGCAGCCGAACGACCGAGCGCAGCGAGTCAGTGAGCGAGGAAGCGGAAGAGCGCCCAATACGCAAACCGCCTCTCCCCGCGCGTTGGCCG
ATTCATTAATG

pAAV-CMV-TYR_STOP Sequence

CAGCAGCTGCGCGCTCGCTCGCTCACTGAGGCCGCCCGGGCAAAGCCCGGGCGTCGGGCGACCTTTGGTCGCCCGGCCTCAGTGAGCGAGCGAGCGCG
CAGAGAGGGAGTGGCCAACTCCATCACTAGGGGTTCCTTGTAGTTAATGATTAACCCGCCATGCTACTTATCTACGTAGCCATGCTCTAGACATGGCTCGACA
GATCTCAATATTGGCCATTAGCCATATTATTCATTGGTTATATAGCATAAATCAATATTGGCTATTGGCCATTGCATACGTTGTATCTATATCATAATATGTACATTTAT
ATTGGCTCATGTCCAATATGACCGCCATGTTGGCATTGATTATTGACTAGTTATTAATAGTAATCAATTACGGGGTCATTAGTTCATAGCCCATATATGGAGTTCCG
CGTTACATAACTTACGGTAAATGGCCCGCCTGGCTGACCGCCCAACGACCCCCGCCCATTGACGTCAATAATGACGTATGTTCCCATAGTAACGCCAATAGGG
ACTTTCCATTGACGTCAATGGGTGGAGTATTTACGGTAAACTGCCCACTTGGCAGTACATCAAGTGTATCATATGCCAAGTCCGCCCCCTATTGACGTCAATGA
CGGTAAATGGCCCGCCTGGCATTATGCCCAGTACATGACCTTACGGGACTTTCCTACTTGGCAGTACATCTACGTATTAGTCATCGCTATTACCATGGTGATGC
GGTTTTGGCAGTACACCAATGGGCGTGGATAGCGGTTTG ACTCACGGGGATTTCCAAGTCTCCACCCCATTGACGTCAATGGGAGTTTGTTTTGGCACCAAA
ATCAACGGGACTTTCCAAAATGTCGTAACAACTGCGATCGCCCGCCCCGTTGACGCAAATGGGCGGTAGGCGTGTACGGTGGGAGGTCTATATAAGCAGAGC
TCGTTTAGTGAACCGTCAGATCACTAGAAGCTTTATTGCGGTAGTTTATCACAGTTAAATTGCTAACGCAGTCAGTGCTTCTGACACAACA GTCTCGAACTTAA
GCTGCAGTGACTCTCTTAAGGTAGCCTTGCAGAAGTTGGTCGTGAGGCACTGGGCAGGTAAGTATCAAGGTTACAAGACAGGTTTAAGGAGACCAATAGAAA
CTGGGCTTGTCGAGACAGAGAAGACTCTTGCGTTTCTGATAGGCACCTATTGGTCTTACTGACATCCACTTTGCCTTTCTCTCCACAGGTGTCCACTCCCAGT
TCAATTACAGCTCTTAAGGCTAGAGTACTTAATACGACTCACTATAGGCTAGCCTCGACCTCGAGACGCGTGATAAACTTAAGCTTGGTACCGAGCTCGGATCC
ACTAGTCCAGTGTGGTGGAATTCTGCAGATATCCAGCACAGTGGCGGCCGCTCGACCGACCTTGTGAGGACTAGAGGAAGAATGCTCCTGGCTGTTTTGTAC
TGCCTGCTGTGGAGTTTCCAGACCTAAGCTGGCCATTTCCCTAGAGCCTGTGTCTCCTAAAAGAACCTGATGGAGAAGGAATGCTGAC CACCGTGGAGCGG
GGACAGGAGTCCCTGTGGCCAGCTTTCAGGCAGAGGTTCCTGTCAGAATATCCTTCTGTCCAATGCACCACTTGGGCCTCAATTTCCCTTCACAGGGGTGGA
TGACCGGGAGTCGTGGCCTTCCGTCTTTTATAATAGGACCTGCCAGTGCTCTGGCAACTTCATGGGATTCAACTGTGGAAACTGCAAGTTTGGCTTTTGGGG
ACCAAACTGCACAGAGAGACGACTCTTGGTGAGAAGAAA CATCTTCGATTTGAGTGCCCCAGAGAAGGACAAATTTTTTGCCTACCTCACTTTAGCAAAGCAT
ACCATCAGCTCAGACTATGTCATCCCCATAGGGACCTATGGCCAAATGAAAAATGGATCAACACCCATGTTTAACGACATCAATATTTATGACCTCTTTGTCTGG
ATGCATTATTATGTGTCAATGGATGCACTGCTTGGGGGATCTGAAATCTGGAGAGACATTGATTTTGCCCATGAAGCACCAGCTTTT CTGCCTTGGCATAGACT
CTTCTTGTTGCGGTGGGAACAAGAAATCCAGAAGCTGACAGGAGATGAAAACTTCACTATTCCATATTGGGACTGGCGGGATGCAGAAAAGTGTGACATTTGC
ACAGATGAGTACATGGGAGGTCAGCACCCCACAAATCCTAACTTACTCAGCCCAGCATCATTCTTCTCCTCTTGGCAGATTGTCTGTAGCCGATTGGAGGAGT
ACAACAGCCATCAGTCTTTATGCAATGGAACGCCCGAGGGACCTTTACGGCGTAATCCTGGAAACCATGACAAATCCAGAACCCCAAGGCTCCCCTCTTCAG
CTGATGTAGAATTTTGCCTGAGTTTGACCCAATATGAATCTGGTTCCATGGATAAAGCTGCCAATTTCAGCTTTAGAAATACACTGGAAGGATTTGCTAGTCCAC
TTACTGGGATAGCGGATGCCTCTCAAAGCAGCATGCACAATGCCTTGCACATCTATATGAATGGAACAATGTCCCAGGTACAGGGATCTGCCAACGATCCTATC
TTCCTTCTTCACCATGCATTTGTTGACAGTATTTTTGAGCAGTGGCTCCGAAGGCACCGTCCTCTTCAAGAAGTTTATCCAGAAGCCAATGCACCCATTGGACA
TAACCGGGAATCCTACATGGTTCCTTTTATACCACTGTACAGAAATGGTGATTTCTTTATTTCATCCAAAGATCTGGGCTATGACTATAGCTATCTACAAGATTCAG
ACCCAGACTCTTTTCAAGACTAC ATTAAGTCCTATTTGGAACAAGCGAGTCGGATCTGGTCATGGCTCCTTGGGGCGGCGATGGTAGGGGCCGTCCTCACTG
CCCTGCTGGCAGGGCTTGTGAGCTTGCTGTGTCGTCACAAGAGAAAGCAGCTTCCTGAAGAAAAGCAGCCACTCCTCATGGAGAAAGAGGATTACCACAGC
TTGTATCAGAGCCATTTATAAAAGGCTTAGGCAATAGAGTAGGGCCAAAAAGCCTGACCTCACTCTAACTCAAAGTAATGTCCAGGTTCCCAGAGAATATCTGC
TGGTATTTTTCTGTAAAGACCATTTGCAAAATTGTAACCTAATACAAAGTGTAGCCTTCTTCCAACTCAGGTAGAACACACCTGTCTTTGTCTTGCTGTTTTCACT
CAGCCCTTTTAACATTTTCCCCTAAGCCCCTAGAGGGCCCGTTTATCGGATCCCGGCCGGCGGCCGCTTCCCTTTAGTGAGGGTTAATGCTTCGAGCAGACA
TGATAAGATACATTGATGAGTTTGGACAAACCACAACTAGAATGCAGTGAAAAAAATGCTTTATTTGTGAAATTTGTGATGCTATTGCTTTATTTGTAACCATTATA
AGCTGCAATAAACAAGTTAACAACAACAATTGCATTCATTTTATGTTTCAGGTTCAGGGGGAGATGTGGGAGGTTTTTTAAAGCAAGTAAAACCTCTACAAATGT
GGTAAAATCCGATAAGGACTAGAGCATGGCTACGTAGATAAGTAGCATGGCGGGTTAATCATTAACTACAAGGAACCCCTAGTGATGGAGTTGGCCACTCCC
TCTCTGCGCGCTCGCTCGCTCACTGAGGCCGGGCGACCAAAGGTCGCCCGACGCCCGGGCTTTGCCCGGGCGGCCTCAGTGAGCGAGCGAGCGCGCCA
GCTGGCGTAATAGCGAAGAGGCCCGCACCGATCGCCCTTCCCAACAGTTGCGCAGCCTGAATGGCGAATGGAATTCCAGACGATTGAGCGTCAAAATGTAG
GTATTTCCATGAGCGTTT TTCCGTTGCAATGGCTGGCGGTAATATTGTTCTGGATATTACCAGCAAGGCCGATAGTTTGAGTTCTTCTACTCAGGCAAGTGATGT
TATTACTAATCAAAGAAGTATTGCGACAACGGTTAATTTGCGTGATGGACAGACTCTTTTACTCGGTGGCCTCACTGATTATAAAAACACTTCTCAGGATTCTGG
CGTACCGTTCCTGTCTAAAATCCCTTTAATCGGCCTCCTGTTTAGCTCCCGCTCTGATTCTAAC GAGGAAAGCACGTTATACGTGCTCGTCAAAGCAACCATAG
TACGCGCCCTGTAGCGGCGCATTAAGCGCGGCGGGTGTGGTGGTTACGCGCAGCGTGACCGCTACACTTGCCAGCGCCCTAGCGCCCGCTCCTTTCGCTT
TCTTCCCTTCCTTTCTCGCCACGTTCGCCGGCTTTCCCCGTCAAGCTCTAAATCGGGGGCTCCCTTTAGGGTTCCGATTTAGTGCTTTACGGCACCTCGACC
CCAAAAAACTTGATTAGGGTGATGGTTCACGTAGTGGGCCATCGCCCTGATAGACGGTTTTTCGCCCTTTGACGTTGGAGTCCACGTTCTTTAATAGTGGACT
CTTGTTCCAAACTGGAACAACACTCAACCCTATCTCGGTCTATTCTTTTGATTTATAAGGGATTTTGCCGATTTCGGCCTATTGGTTAAAAAATGAGCTGATTTAA
CAAAAATTTAACGCGAATTTTAACAAAATATTAACGTCTACAATTTAAATATTTGCTTATACAATCTTCCTGTTTTTGGGGCTTTTCTGATTATCAACCGGGGTACAT
ATGATTGACATGCTAGTTTTACGATTACCGTTCATCGATTCTCTTGTTTGCTCCAGACTCTCAGGCAATGACCTGATAGCCTTTGTAGAGACCTCTCAAAAATAG
CTACCCTCTCCGGCATGAATTTATCAGCTAGAACGGTTGAATATCATATTGATGGTGATTTGACTGTCTCCGGCCTTTCTCACCCGTTTGAATCTTTACCTACA C
ATTACTCAGGCATTGCATTTAAAATATATGAGGGTTCTAAAAATTTTTATCCTTGCGTTGAAATAAAGGCTTCTCCCGCAAAAGTATTACAGGGTCATAATGTTTTT
GGTACAACCGATTTAGCTTTATGCTCTGAGGCTTTATTGCTTAATTTTGCTAATTCTTTGCCTTGCCTGTATGATTTATTGGATGTTGGAATCGCCTGATGCGGTA
TTTTCTCCTTACGCATCTGTGCGGTATTTCACACCGCATATG GTGCACTCTCAGTACAATCTGCTCTGATGCCGCATAGTTAAGCCAGCCCCGACACCCGCCAA
CACCCGCTGACGCGCCCTGACGGGCTTGTCTGCTCCCGGCATCCGCTTACAGACAAGCTGTGACCGTCTCCGGGAGCTGCATGTGTCAGAGGTTTTCACC
GTCATCACCGAAACGCGCGAGACGAAAGGGCCTCGTGATACGCCTATTTTTATAGGTTAATGTCATGATAATAATGGTTTCTTAGACGTCAGGT GGCACTTTTC
GGGGAAATGTGCGCGGAACCCCTATTTGTTTATTTTTCTAAATACATTCAAATATGTATCCGCTCATGAGACAATAACCCTGATAAATGCTTCAATAATATTGAAAA
AGGAAGAGTATGAGTATTCAACATTTCCGTGTCGCCCTTATTCCCTTTTTTGCGGCATTTTGCCTTCCTGTTTTTGCTCACCCAGAAACGCTGGTGAAAGTAAA
AGATGCTGAAGATCAGTTGGGTGCACGAGTGGGTT ACATCGAACTGGATCTCAACAGCGGTAAGATCCTTGAGAGTTTTCGCCCCGAAGAACGTTTTCCAAT
GATGAGCACTTTTAAAGTTCTGCTATGTGGCGCGGTATTATCCCGTATTGACGCCGGGCAAGAGCAACTCGGTCGCCGCATACACTATTCTCAGAATGACTTG
GTTGAGTACTCACCAGTCACAGAAAAGCATCTTACGGATGGCATGACAGTAAGAGAATTATGCAGTGCTGCCATAACCATGAGTGATAACACTGCGGCCAACT
TACTTCTGACAACGATCGGAGGACCGAAGGAGCTAACCGCTTTTTTGCACAACATGGGGGATCATGTAACTCGCCTTGATCGTTGGGAACCGGAGCTGAATG
AAGCCATACCAAACGACGAGCGTGACACCACGATGCCTGTAGCAATGGCAACAACGTTGCGCAAACTATTAACTGGCGAACTACTTACTCTAGCTTCCCGGCA
ACAATTAATAGACTGGATGGAGGCGGATAAAGTTGCAGGACCACTTCTGCGCTCGGCCCTTCCGGCTGGCTGGTTTATTGCTGATAAATCTGGAGCCGGTGA
GCGTGGGTCTCGCGGTATCATTGCAGCACTGGGGCCAGATGGTAAGCCCTCCCGTATCGTAGTTATCTACACGACGGGGAGTCAGGCAACTATGGATGAACG
AAATAGACAGATCGCTGAGATAGGTGCCTCACTGATTAAGCATTGGTAACTGTCAGACCAAGTTTACTCATATATACTTTAGATTGATTTAAAACTTCATTTTTAAT
TTAAAAGGATCTAGGTGAAGATCCTTTTTGATAATCTCATGACCAAAATCCCTTAACGTGAGTTTTCGTTCCACTGAGCGTCAGACCCCGTAGAAAAGATCAAA
GGATCTTCTTGAGATCCTTTTTTTCTGCGCGTAATCTGCTGCTTGCAAACAAAAAAACCACCGCTACCAGCGGTGGTTTGTTTGCCGGATCAAGAGCTACCAA
CTCTTTTTCCGAAGGTAACTGGCTTCAG CAGAGCGCAGATACCAAATACTGTCCTTCTAGTGTAGCCGTAGTTAGGCCACCACTTCAAGAACTCTGTAGCACC
GCCTACATACCTCGCTCTGCTAATCCTGTTACCAGTGGCTGCTGCCAGTGGCGATAAGTCGTGTCTTACCGGGTTGGACTCAAGACGATAGTTACCGGATAAG
GCGCAGCGGTCGGGCTGAACGGGGGGTTCGTGCACACAGCCCAGCTTGGAGCGAACGACCTACACCGAACTGAGATAC CTACAGCGTGAGCTATGAGAAA
GCGCCACGCTTCCCGAAGGGAGAAAGGCGGACAGGTATCCGGTAAGCGGCAGGGTCGGAACAGGAGAGCGCACGAGGGAGCTTCCAGGGGGAAACGCC
TGGTATCTTTATAGTCCTGTCGGGTTTCGCCACCTCTGACTTGAGCGTCGATTTTTGTGATGCTCGTCAGGGGGGCGGAGCCTATGGAAAAACGCCAGCAAC
GCGGCCTTTTTACGGTTCCTGGCCTTTTGCTGGC CTTTTGCTCACATGTTCTTTCCTGCGTTATCCCCTGATTCTGTGGATAACCGTATTACCGCCTTTGAGTG
AGCTGATACCGCTCGCCGCAGCCGAACGACCGAGCGCAGCGAGTCAGTGAGCGAGGAAGCGGAAGAGCGCCCAATACGCAAACCGCCTCTCCCCGCGCG
TTGGCCGATTCATTAATG

SUPPLEMENTARY FIGURE 1: Plasmid maps and sequences for pAAV-CMV-hTyr and pAAV-CMV-hTyr_STOP

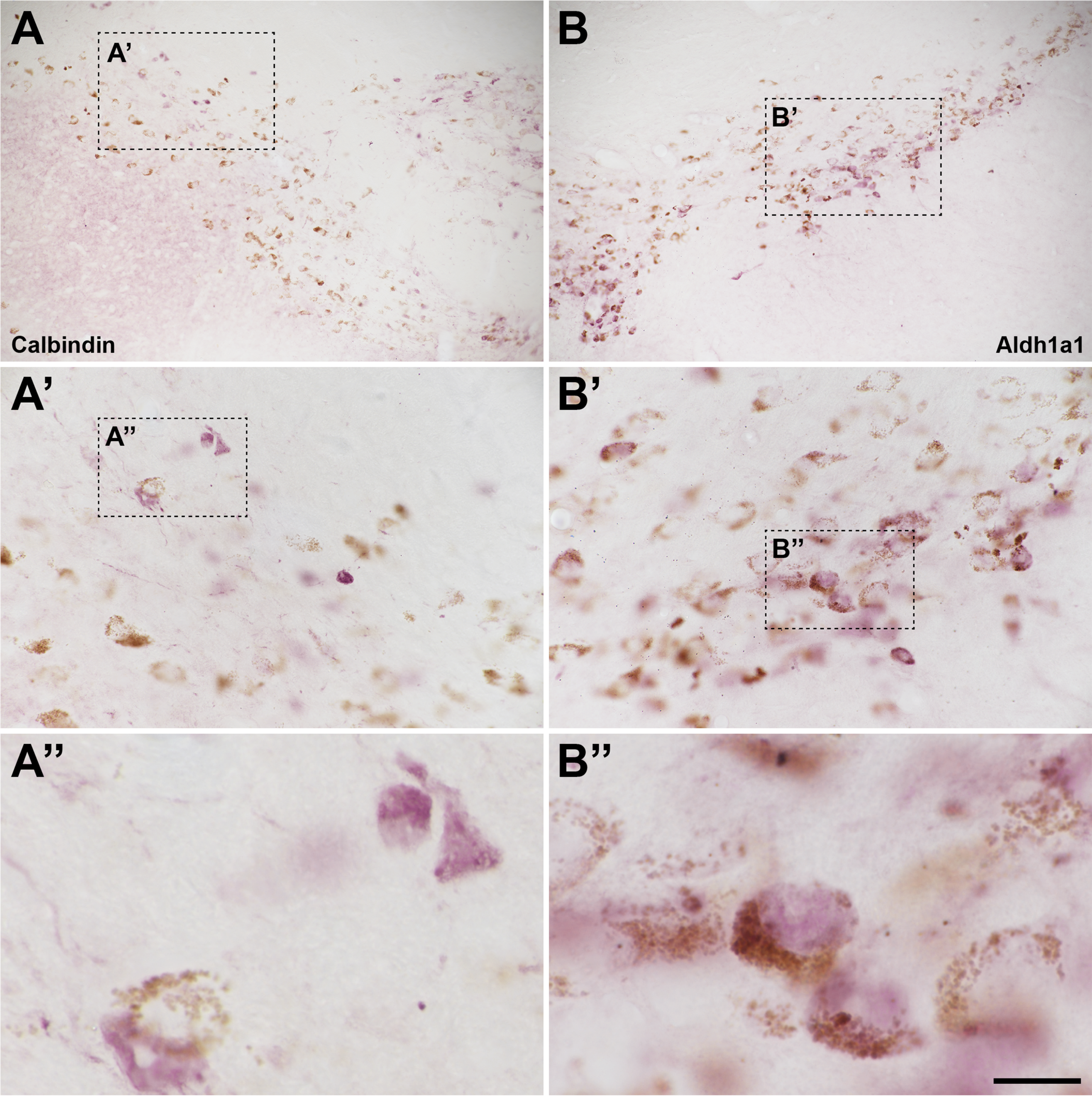

SUPPLEMENTARY FIGURE 2: Tier-specific pigmentation of dopaminergic neurons. The systemic delivery of AAV9-P31-hTyr resulted in a selective pigmentation of dopaminergic neurons located in the ventral tier of the SNpc, as defined by immunohistochemistry for the aldehyde dehydrogenase type 1a1 (Aldh1a1+ neurons). By contrast, calbindin-positive neurons in the dorsal tier of the SNpc never became pigmented. The selective pigmentation of Aldh1a1+ neurons suggested a mechanistic link between neuromelanin accumulation and dopaminergic cell vulnerability. Scale bar, 150 mm (panels A and B); 50 mm (A' and B'); and 15 mm (A'' and B'')

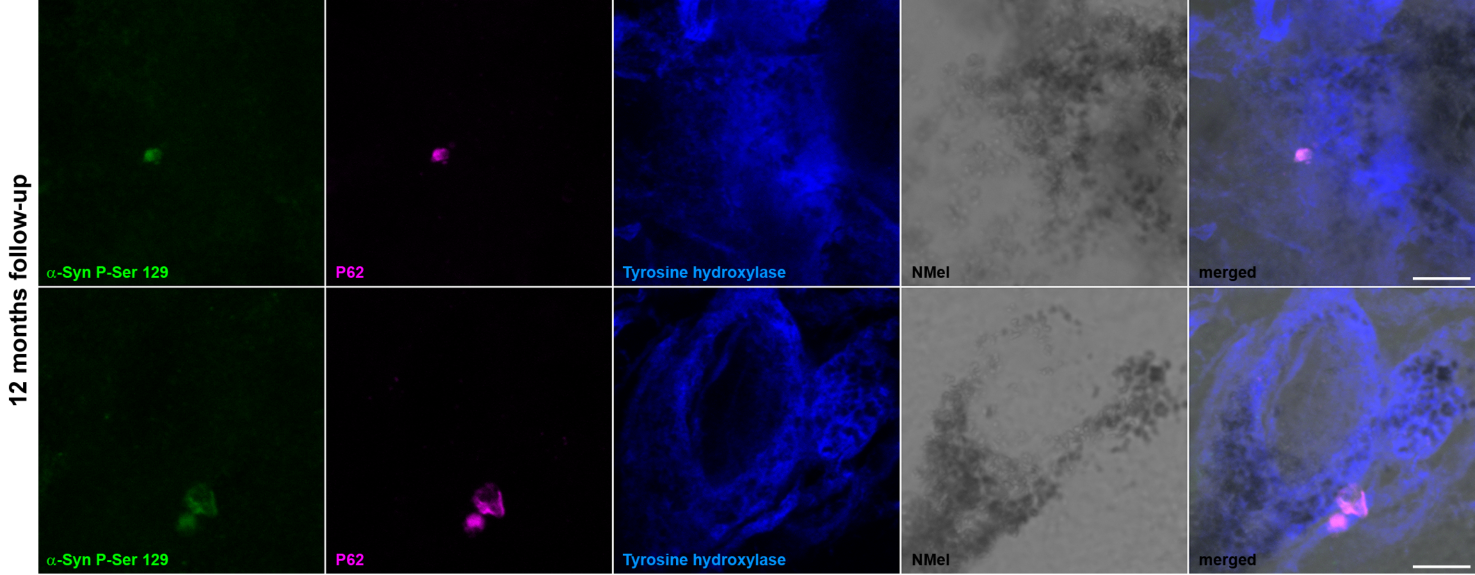

SUPPLEMENTARY FIGURE 3: Lewy body-like inclusions in LC neurons. Intracellular inclusions within noradrenergic neurons of the LC (TH+; blue channel) were only observed twelve months post-delivery of AAV9-P31-hTyr, in parallel to pigmentation levels. Observed intracytoplasmic aggregates are positive for phosphorylated alpha-synuclein (P-Ser 129; green channel) and P62 (purple channel). Scale bars, 5 μm.

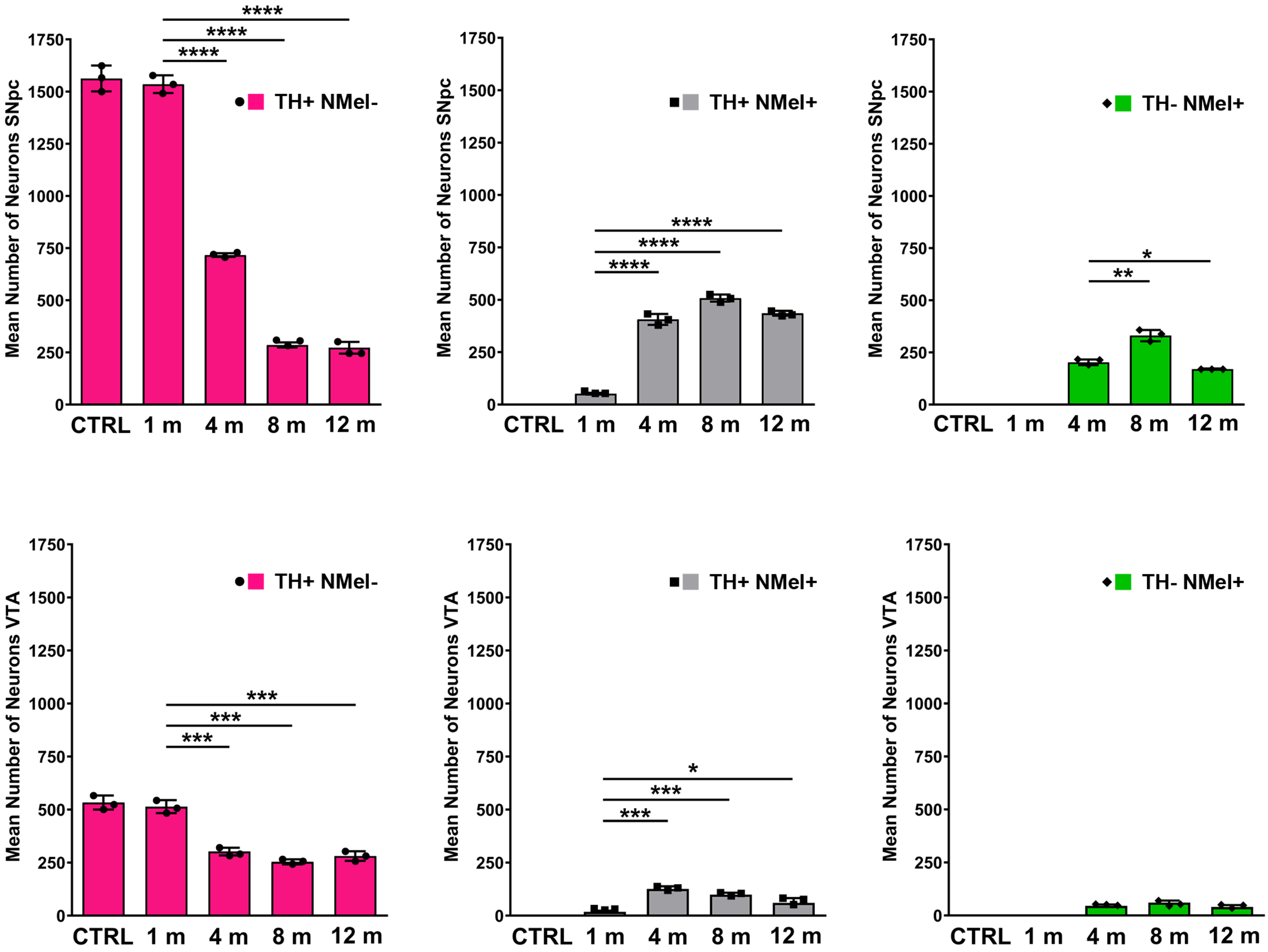

SUPPLEMENTARY FIGURE 4: Phenotype-specific changes in dopaminergic neurons. Histograms showing the time-dependent changes observed for each characteristic cell phenotype in the SNpc and VTA. A major reduction in TH+ / NMel – neurons was observed at four, eight, and twelve months compared to the control group in the SNpc and the VTA. The number of pigmented dopaminergic neurons (TH+ / NMel+) increased over time, reaching a maximum peak at eight months. The same trend applied to ghost neurons (TH- / NMel+) at the level of the SNpc (numbers too low for comparison purposes in the VTA). Data are represented as mean +/- SEM, unpaired t-test. $p < 0.0001$ (TH+ / NMel- neurons in the SNpc; all time points); $p < 0.0001$ (TH+ / NMel + neurons in the SNpc); $p = 0.0018$ (ghost cells, four vs. eight months, SNpc); $p = 0.0155$ (ghost cells; four vs. twelve months, SNpc); p = 0.0005, 0.0002 and 0.004 (TH+ / NMel- neurons, four, eight and twelve vs. one month, respectively, VTA); $p = 0.0001$ (TH+ / NMel- neurons, four and eight months vs. one month, VTA); $p = 0.0362$ (twelve vs. one month, VTA).

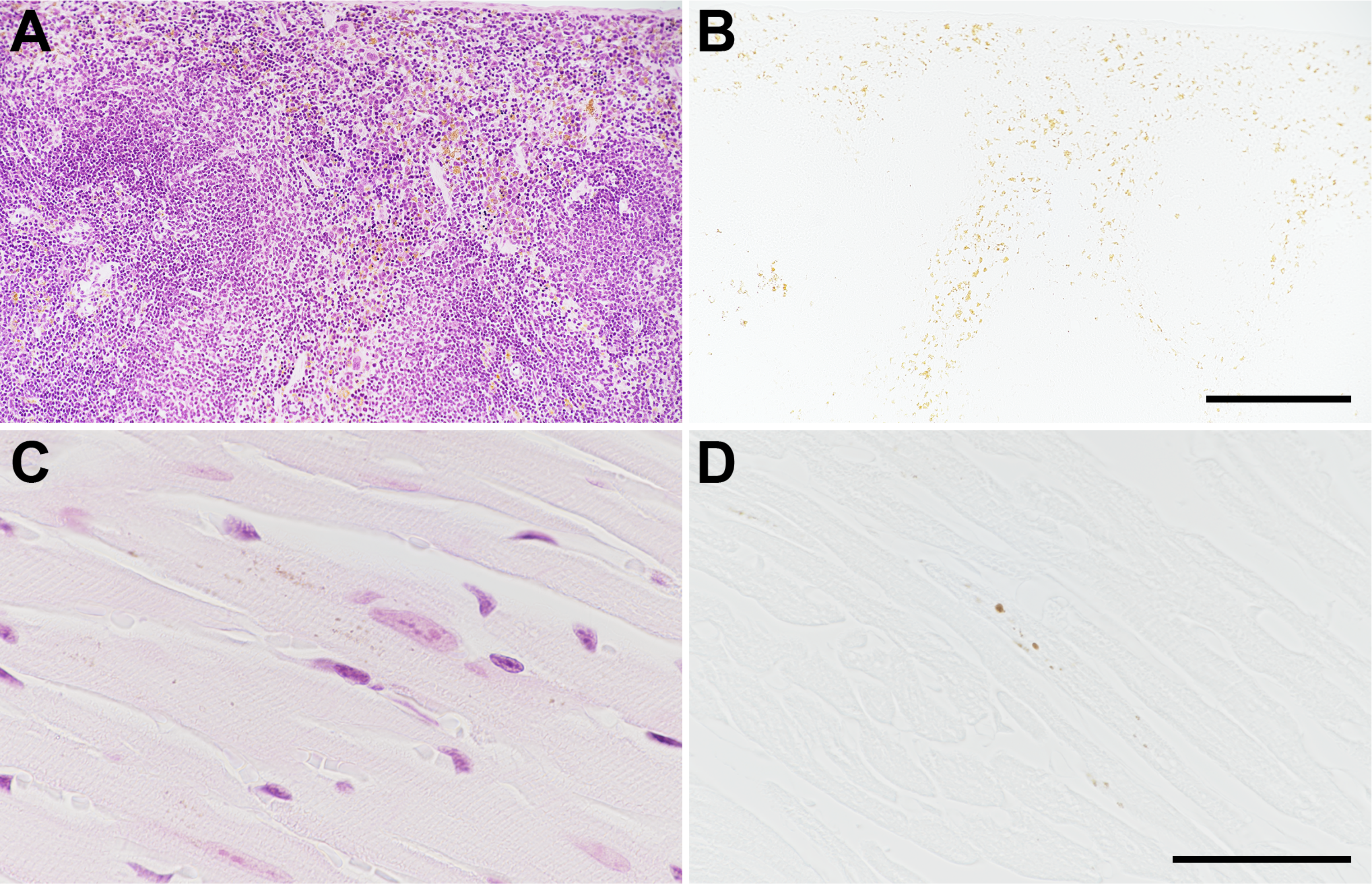

SUPPLEMENTARY FIGURE 5: Pigmented peripheral organs. Following the examination of all peripheral organs extracted during animal necropsies, conducted stains revealed small traces of NMel accumulation in the red pulp of the spleen to a much lower extent in the sarcoplasmic cones of cardiomyocytes beyond four months post-systemic deliveries of AAV9-P31-hTyr. (A & B) Representative photomicrographs taken from the spleen (H&E stained and non-stained, respectively). Scale bar, 200 m. (C & D) NMel accumulation in cardiomyocytes (H&E stained and non-stained, respectively). Scale bar, 40 m. Pigmentation was never observed in peripheral organs other than the spleen and heart
